# Water quality index prediction via a robust machine learning model using oxygen-related indices for river water quality monitoring

**DOI:** 10.1038/s41598-026-36156-3

**Published:** 2026-01-24

**Authors:** Amin Arzhangi, Sadegh Partani

**Affiliations:** Faculty of Engineering, Civil Engineering Department, University of Bojnord, Northern Khorasan, Iran

**Keywords:** Water quality index (WQI), Support vector regression (SVR), reaeration coefficient (K_2_), Biological oxygen demand (BOD), Chemical oxygen demand (COD), Environmental monitoring, River water quality, Oxygen-related indices, Environmental sciences, Hydrology, Water resources

## Abstract

Rivers face increasing pollution, requiring accurate water quality assessment tools. Existing indices like the Water Quality Index (WQI) often overlook the integration of oxygen-related parameters critical to aquatic health. Here, we develop a machine learning model using Support Vector Regression (SVR) to predict the Water Quality Index (WQI_OIs_) by integrating key oxygen-related parameters, including Biological Oxygen Demand (BOD), Chemical Oxygen Demand (COD), Dissolved Oxygen (DO), and the reaeration coefficients (K_1_, K_2_). Applied to three rivers in Iran, the model demonstrated high accuracy, with a cross-validated R² > 0.95 and root mean squared error (RMSE) of 0.92 for the Haraz River and 1.41 for the Simineh River. Predictions showed strong correlation (*r* = 0.98) with standard indices, and feature importance analysis revealed DO as the most influential parameter. The model’s generalizability was confirmed through validation on independent river datasets, highlighting its robustness across diverse hydrological conditions. This approach offers a scalable, interpretable framework for continuous water quality monitoring, enabling more precise and data-driven management of aquatic ecosystems, particularly in regions with varying environmental factors.

**Supplementary Information:**

The online version contains supplementary material available at 10.1038/s41598-026-36156-3.

## Introduction

Various indices have been developed to assess the quality of freshwater, each focusing on different aspects of water health. Key examples include the Water Quality Index (WQI), Biodegradability Index (BI)^1,2^, deoxygenation constant (K_1_), and the reaeration coefficient (K_2_)^3^. While each of these indices provides valuable insights, the WQI remains the most comprehensive, offering a holistic assessment of overall water quality^4^.

Given its significance, numerous studies have introduced alternative equations of the WQI, often employing specific methodologies for calculation or incorporating unique variables in the equation. Additionally, some research has focused on predicting water quality by considering factors such as hydrological and hydraulic conditions^3,5^, climate variables, and other environmental indices, aiming to provide a more integrated understanding of freshwater systems.

House et al. (1989)^6^ developed new water quality indices that integrate legally adopted standards, criteria, and information on potential water use and toxic determinants, allowing for the assessment of changes in a river’s economic potential through shifts in water quality. The General WQI was applied to long-term data series to detect trends, with the lowest determined ratings identifying specific pollutants responsible for quality deterioration. A 5th percentile WQI score, along with 90% confidence limits, was utilized to improve the accuracy of the index in reflecting both water quality changes and potential water use.

The water quality of Ravi River, a tributary of the Indus River, was assessed using the WQI. The WQI consolidates multiple water quality parameters into a single value, making complex data accessible to the public. Eight key parameters, pH, TDS, total hardness, calcium (Ca), magnesium (Mg), alkalinity, dissolved oxygen (DO)^7^, and electrical conductivity (EC), were used to calculate the index. WQI values for River Ravi ranged from 54.8 to 97.88, indicating generally good water quality, with some declines during certain months, especially due to human activities like dam operations. The study concluded that WQI effectively compares water quality across sources and serves as a useful tool for communicating water quality trends to the public and policymakers^8^.

According to Bora and Goswami, (2017)^9^, the Kolong River in Nagaon district, Assam, has undergone significant degradation due to human interventions, particularly the construction of an embankment in 1964 for flood control. Once a thriving distributary of the Brahmaputra River, the river now faces severe ecological decline, with lentic pools of polluted water and minimal socio-economic value. The Central Pollution Control Board has listed the Kolong River among India’s 275 most polluted rivers. The study assessed the seasonal water quality of river using the WQI. Results showed very poor water quality across all seven sampling sites, with the most deterioration observed during the monsoon season (average WQI of 122.47), compared to pre-monsoon (85.73) and post-monsoon (80.75) seasons. Hatimura and Nagaon Towns were identified as the most polluted sites.

Chabuk et al., (2020)^10^ assessed the water quality of the Tigris River using the WQI method and GIS software. Twelve parameters (Ca, Mg, Na, K, Cl, SO4, HCO3, TH, TDS, BOD_5_, NO_3_, and EC) were analyzed at 14 stations along the river. The weighted arithmetic method was used to compute the WQI, while the IDW interpolation method in ArcGIS 10.5 generated prediction maps for the parameters during the wet and dry seasons of 2016. Regression analysis between observed and predicted values showed acceptable determination coefficients (R²). The study revealed water quality degradation in downstream, during both seasons, with the most significant deterioration observed at Qurnah in southern Iraq. The comprehensive scope of study provides valuable insights into the contamination in the river, aiding in the development of appropriate solutions.

Khan et al., (2023)^11^ assessed the water quality of the Jamuna River in Bangladesh at five sites during wet and dry seasons, using six global WQIs to compare the results with Bangladesh’s Environmental Quality Standard (EQS) and Department of Environment (DoE) criteria. The WQI models employed include Weighted Arithmetic WQI (WAWQI), British Columbia WQI (BCWQI), Canadian Council of Ministers of the Environment WQI (CWQI), Assigned WQI (AWQI), Malaysian WQI (MWQI), and Oregon WQI (OWQI), analyzing 15 physicochemical parameters. Findings show that most parameters exceed permissible values, with average water quality across sites falling into the lowest category. Correlations were observed between WAWQI and AWQI, as well as MWQI and OWQI. The river water, while unsuitable for household and drinking use, is fit for irrigation. Human activities, urbanization, and industrial waste are likely contributing to the water quality decline. Regular monitoring and regular measures are essential to restore acceptable water standards.

Das (2025)^12^ assessed post-monsoon water quality of the Baitarani River, Odisha, using WQI at 13 sites (2021–2024). Turbidity exceeded drinking limits at all sites. WAWQI, CWQI, and Integrated Weight (IWQI) indicated that 54–77% of sites were poorly polluted. Cluster and principal component analyses identified natural processes, agriculture, municipal, and industrial discharges as major pollution sources. The study highlights the need to reduce sewage, stormwater, and solid waste inputs to improve river water quality.

Given importance WQI, considerable research has focused on simplifying and predicting the various WQI. Numerous studies have successfully applied various machine learning (ML) models such as artificial neural networks (ANN)^13^, support vector machines (SVR)^14^, and tree-based ensembles to predict WQI using a range of physicochemical parameters, often demonstrating superior accuracy over traditional statistical methods. These models have proven effective in diverse geographical contexts, from the Ravi River in India to the Tigris River in Iraq. However, these studies typically rely on a broad suite of input parameters that may not be consistently or easily available, limiting the universal applicability of their models. While some studies have explored the relationships between WQI and individual parameters such as DO^15^, DO deficit^16^, BOD^17^, or COD^18^, most focus on single variables, with few considering the combined influence of nutrients, coliforms, and other water quality factors. To date, no study has utilized oxygen-related indices collectively to derive comprehensive WQI values.

This study postulates that oxygen-related indices, including BOD, COD, DO, K_1_, K_2_, and DO deficit, effectively reflect specific aspects of water quality. For example, BOD and COD indicate biological and chemical pollution respectively, K_2_ reflects the river’s self-purification capacity, and K_1_ represents conditions that may amplify pollution. By integrating these indices, this research proposes a unified oxygen indices WQI (WQI_OIs_) that captures multiple dimensions of water quality. The primary innovation of this study lies in addressing this gap through a holistic framework that combines three key novel aspects. First, we move beyond single-river, site-specific models by developing and validating a single, universally applicable “Super Model” designed for robust performance across diverse and unseen river ecosystems. Second, we pioneer a minimalist approach by demonstrating that a comprehensive WQI can be accurately predicted using only a core set of four universally available oxygen-related indices (DO, BOD, COD, and Temperature), significantly enhancing the model’s practicality and cost-effectiveness for real-world application. Finally, we emphasize model transparency by integrating advanced interpretability techniques (SHAP) to ensure our model is not an opaque “black box,” but an ecologically coherent tool whose decision-making process can be trusted by environmental managers. Together, these contributions establish a new, scalable, and trustworthy paradigm for water quality assessment.

## Materials and methods

### Study area

**Fig. 1:**
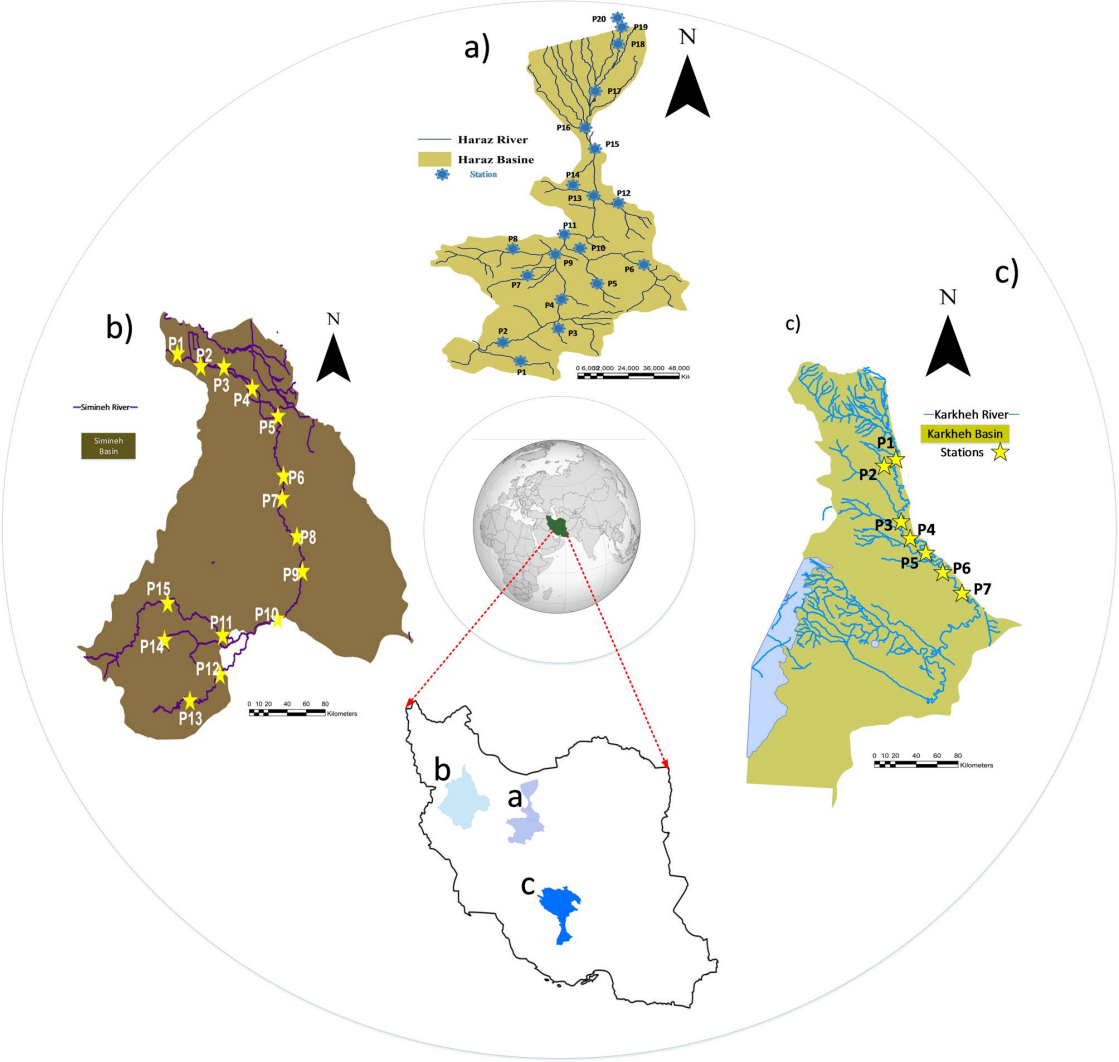
case studies including **(a)** Simineh River **(b)** Haraz River **(c)** Karkheh River.

This study focuses on three major rivers in Iran, the Karkheh, Haraz, and Simineh, which were strategically selected to serve as a robust testbed for model development and validation (Fig. 1). The primary justification for their selection is their significant diversity in hydro-climatic and environmental conditions. The Karkheh River flows through semi-arid regions, the Haraz is situated in a temperate, mountainous area, and the Simineh drains a sub-catchment of the environmentally sensitive Lake Urmia watershed. This diversity provides a wide spectrum of water quality conditions, making it an ideal setting to develop a single, universally applicable “Super Model” and rigorously test its ability to generalize across different ecosystem types, a core objective of this research.

The Karkheh River, the third-longest river in Iran after the Karun and Sefidroud rivers, originates from the Zagros Mountains and flows through the northern part of Khuzestan, particularly near Pol-Zal. The river is formed by the confluence of the Pol-Zal and Seymare rivers near Andimeshk and Susa. largest dam in Iran, the Karkheh Dam, is located on this river^19^. The Karkheh basin experiences a wide range of climatic conditions, with temperatures recorded from − 25 °C to + 50 °C. The basin has a Mediterranean climate, with an average annual precipitation of approximately 290.6 mm at the dam site, and a mean annual temperature of about 24.6 °C, with recorded extremes of 53.6 °C and − 4.2 °C.

The Simineh River basin is a significant sub-catchment of the Lake Urmia watershed, located in the northwestern parts of Iran, specifically in the West Azerbaijan and Kurdistan provinces^20^. The river originates from the highlands south and southwest of Saqqez County, flowing through mountainous terrain and plains before entering the Miandoab plain and eventually draining into Lake Urmia. The basin exhibits notable topographic and climatic diversity, with elevations ranging from 1,352 m at the headwaters to approximately 1,200 m in the Miandoab plain. The basin encompasses parts of Saqqez, Bukan, Mahabad, and Miandoab counties, covering an area of 3,787 km². Geologically, the basin is characterized by carbonate and non-carbonate sedimentary formations, as well as volcanic and metamorphic units. Land use in the area is dominated by agriculture, with the remainder consisting of rangelands, forests, and human settlements. The Simineh River is crucial for drinking water, irrigation, and groundwater recharge, although it faces challenges from urban expansion, industrial discharges, and agricultural runoff, which have significantly degraded its water quality. Consequently, the Simineh River basin is an important case study in water resource management and environmental challenges in semi-arid regions of Iran.

The study area also includes the northern part of Iran, covering a significant portion of the southern coastal plain of the Caspian Sea. This region is defined by the watersheds of the Haraz, Garmarud, and Babolrud rivers. Geographically, the area extends from the Caspian Sea coastline in the north to the central Alborz mountain range in the south. The Haraz River system dominates the hydrology of the region, originating from Mount Damavand and draining a watershed of approximately 4,000 km². Two main tributaries of river, the Noor and Lar rivers, converge south of Amol, and the river flows northwards into the Caspian Sea. The region is water-rich^21^, characterized by a high density of rivers and streams fed by mountain precipitation and snowmelt, although seasonal flow variations are significant. The region is water-rich, characterized by a high density of rivers and streams fed by mountain precipitation and snowmelt, although seasonal flow variations are significant. Annual precipitation averages around 770–850 mm, with the highest levels occurring in autumn and winter. The average annual temperature is approximately 14.1 °C, with clear seasonal variations, the warmest month being August and the coolest being February, Relative humidity is consistently high, contributing to the region’s lush vegetation.

Human activities have significantly shaped the landscape, with agriculture covering approximately 46.1% of the area, predominantly in the fertile coastal plains. Forests make up 29.6% of the land, mainly in the southern mountainous areas, while mixed-use zones of agriculture and orchards account for 18.3%. Urban areas occupy 3.5%, and ecologically significant water reservoirs known as Ab-bandans cover 1.1% of the study area.

### Field procedures and In-Situ measurements

The training dataset for the SVR “Super Model” was sourced from an intensive monitoring program on the Karkheh River. Monthly water samples were systematically collected from January 2022 to November 2022 at seven strategically selected stations. This 11-month period provided a comprehensive dataset covering a full range of seasonal variations. In total, 77 samples (7 stations × 11 months) were collected and analyzed, forming the basis for training the model^22^.

The first independent validation dataset was from the Haraz River basin. Data were collected in a single, comprehensive sampling campaign across 20 strategically chosen stations. This resulted in a total of 20 samples, providing a spatially diverse snapshot for the first validation test.

The second validation dataset was from a monitoring program on the Simineh River. Sampling was conducted seasonally at 15 primary stations over a one-year period, comprising a total of four sampling rounds (one per season). This resulted in a dataset of 60 samples (15 stations × 4 rounds), offering a temporally representative dataset to test the model’s generalization capabilities across different seasonal conditions.

The reliability of the water quality assessment in this study was heavily reliant on a meticulously planned sampling and measurement protocol. Monthly water samples were collected from January to November across twenty strategically chosen stations. To minimize the effects of diurnal fluctuations, especially concerning DO levels, all in-situ measurements were consistently conducted between 08:00 and 10:00 AM^23^. At each station, key physicochemical parameters, including water temperature (T_w_), DO, EC, and pH, were measured in-situ using a calibrated YSI ProDSS multi-parameter probe^24^. These measurements were performed before the collection of physical water samples to ensure the data reflected undisturbed, natural stream conditions.

Instrument accuracy was maintained through daily calibration protocols, performed each morning before deployment, using certified standard solutions, in line with manufacturer specifications (e.g., WTW Multi 340i/SET, HORIBA Checker V-10). To improve sample representativeness and minimize surface-level biases, water samples were taken from the mid-depth of the water column (approximately 50% of the total depth) using a Van Dorn horizontal sampler^25^. For increased accuracy and reproducibility, all in-situ measurements were performed in triplicate, with the average value used for subsequent data analysis.

The integrity of the water quality values (WQVs) assessment relied on the precision of the sampling and measurement procedures. Monthly water samples were systematically collected from January 2021 to November 2022 at seven strategically selected stations along the River. These stations represented the micro-basins of the upstream reach, where land-use changes, point-source discharges, and flow rates exhibit minimal variation. A total of 77 samples were collected over the 11-month period. Sampling occurred on the first Monday of each month, between 06:00 and 10:00 AM, to reduce interference from daylight variations, particularly concerning DO concentrations, which are affected by photosynthetic processes^23^.

At each station, in-situ measurements of core physicochemical parameters (T, DO, EC, and pH) were conducted using a calibrated YSI ProDSS multi-parameter probe^24^. To ensure unbiased sampling, water samples were collected from the mid-depth of the river, approximately 50% of the total water column depth, using a Van Dorn horizontal sampler. Additionally, river velocity was measured using a Molina propeller meter (number 4), which has an accuracy of 0.06 and can detect flow rates of up to 5 m/s^24^. To minimize human error in water height (H) measurements, a two-person system was implemented, where both individuals took measurements simultaneously from a perpendicular angle to the water surface.

The monitoring network for the Simineh River was strategically designed to capture spatio-temporal dynamics in water quality across the basin. A total of 15 primary sampling stations were established, extending from the upstream headwaters to the downstream reaches near Miandoab. Station selection followed a multi-criteria approach, prioritizing locations based on key hydrological features (e.g., confluences with major tributaries and sites upstream and downstream of urban centers like Bukan and Miandoab), pollution sources (e.g., downstream of point sources such as the Bukan Wastewater Treatment Plant and non-point sources like agricultural drainage), and logistical considerations for seasonal accessibility and safety.

Sampling was conducted seasonally in four campaigns to capture variability across different hydrological conditions, such as high-flow spring and low-flow autumn. In-situ physicochemical parameters, including T, DO, pH, and EC, were measured at each station using calibrated portable multi-parameter probes (e.g., Crison OXI 45, Hach DR/4000 Spectrophotometer). Water samples for laboratory analysis were collected from the main thalweg at mid-depth to ensure representativeness and avoid surface or benthic biases^26^. This robust sampling framework enabled a comprehensive analysis of pollution transport, attenuation, and self-purification processes within the river system.

### Sample Preservation, Transport, and quality assurance

To maintain the integrity of water samples from collection through to laboratory analysis, stringent preservation and handling procedures were adhered to, following established protocols, including those from the U.S. Environmental Protection Agency (EPA) Region 4 and standard environmental guidelines. Immediately after collection, samples were transferred into pre-cleaned polyethylene bottles and stored in portable iceboxes at approximately 4°^C^ to minimize biological and chemical activity. This temperature control helped preserve reactive parameters such as nutrients and biochemical oxygen demand (BOD_5_). Samples were then transported to the laboratory within 24 h to prevent degradation, ensuring they remained within the prescribed holding times to avoid any changes that could affect the analysis^27^.

Quality assurance and quality control (QA/QC) measures were systematically followed throughout the sampling and transport processes. This included the use of field duplicates to evaluate sampling precision, equipment blanks to check for contamination, and detailed documentation of sampling conditions, including the time, weather, and visual observations. A chain-of-custody protocol was also maintained to ensure sample traceability and preserve the integrity of the data from collection through to analysis. Field activities were meticulously documented, ensuring full transparency and defensibility of the study’s findings^28^.

### Laboratory analytical methods and quality control

Once received in the laboratory, water samples were analyzed using internationally recognized methods to ensure consistent and reliable results. These analyses included gravimetric methods for Total Solids (TS), Total Suspended Solids (TSS), and Total Dissolved Solids (TDS), spectrophotometric methods for nutrients like Nitrate-Nitrogen (NO₃-N) and Orthophosphate (PO₄-P), as well as the BOD_5_^29^ test and microbiological analysis for Total Coliforms (TC) and Fecal Coliforms (FC).

All laboratory analyses followed the procedures outlined in the Standard Methods for the Examination of Water and Wastewater (APHA, 2023), ensuring methodological consistency and comparability. Specific methods included:

- Nutrient analysis using a Seal AQ2 discrete analyzer with detection limits of 0.01–0.05 mg/L.
- Atomic absorption spectrophotometry using a Varian Specter AAA 400 for metal analysis.
- Turbidity measured by a Hach 2100 N turbidimeter.
- TSS determined through gravimetric analysis following Standard Method 2540D.
- BOD₅ measured through the 5-day incubation method at 20 °C.

For quality control, all samples were analyzed in duplicate to assess precision and accuracy. Regular calibration of instruments with certified standards, analysis of method blanks, and use of matrix spikes helped assess analytical recovery and identify any potential interference in the samples. Additional QA/QC measures included external checks, field duplicates analyzed by independent teams, and split samples for comparison with outside laboratories for validation.

A comprehensive Quality Assurance Project Plan (QAPP) was developed to outline all procedures, ensuring high data quality. Field blanks, lab replicates, spike samples, and calibration blanks were incorporated throughout the study to further verify the reliability of the measurements and minimize errors in the analytical process. These rigorous measures were implemented to uphold the integrity of the study and ensure the accuracy of the results.

### Initial assessment of datasets and preliminary correlation analysis to explore parameter relationships

Prior to model training, the consolidated dataset from the Karkheh River underwent a critical preprocessing phase to ensure the optimal performance of the SVR algorithm. This step is essential because SVR is sensitive to the scale and range of the input features; variables with larger magnitudes can disproportionately influence the model’s learning process.

To address this, all input features, DO, BOD, COD, and Water Temperature, were standardized using the Z-score method (Eq. 1). This technique rescales each feature so that it has a mean (µ) of 0 and a standard deviation (σ) of 1. The transformation for each data point x is calculated as:

1 $$\:Z=\frac{X-\mu\:}{\sigma\:}$$

$$z=(x-\upmu)/\sigma$$

The initial assessment of the water quality dataset aimed to understand its inherent structure, variability, and inter-relationships among the measured hydrochemical parameters before proceeding to detailed modeling. Descriptive statistics, including mean, median, standard deviation, minimum, maximum, and range, were first calculated for all parameters to examine data distributions, detect extreme values, and identify missing or censored observations. Missing values and censored data (e.g., values reported as < 1) were appropriately imputed or replaced to maintain numerical consistency.

To visualize the overall structure of the multi-dimensional dataset, Uniform Manifold Approximation and Projection (UMAP) was applied, reducing the dataset to a two-dimensional representation^30^. This approach facilitated the identification of intrinsic patterns and potential clusters, revealing similarities and differences among the sampling sites and rivers.

Outlier detection was performed using a One-Class Support Vector Machine (SVM), complemented by standardized Z-scores computed for each parameter^31^. Heatmaps of these standardized scores were generated to visualize parameter-wise deviations and identify anomalous samples, ensuring that extreme values did not bias further statistical or modeling procedures. In addition, one-way ANOVA was conducted to statistically evaluate differences between inlier and outlier groups, computing F-statistics and p-values for each parameter to identify variables that contributed most to water quality variability^32^.

Collectively, these analyses provided a comprehensive understanding of the dataset’s structure, inter-parameter relationships, and anomalous observations, laying a robust foundation for subsequent ecological interpretation and predictive modeling of water quality dynamics.

### Developing a WQI_OIs_ prediction model using Oxygen-Related indices

Recognizing DO as the paramount indicator of aquatic ecosystem health, we developed a predictive model for the WQI_OIs_ centered on DO dynamics and its key influencing factors. A Support Vector Regression (SVR) model^33^ was selected as the core predictive engine due to its proven efficacy in capturing complex, non-linear relationships, which are characteristic of environmental systems. The development process was structured to create a robust, generalizable, and interpretable model.

The predictive power of an SVR model is highly dependent on the selection of its key hyperparameters. For this study, which utilized the common and effective Radial Basis Function (RBF) kernel, three primary hyperparameters were optimized: C, γ (gamma), and ε (epsilon).

- **The Regularization Parameter (C)**: This parameter controls the trade-off between achieving a low training error and minimizing model complexity (i.e., keeping the decision surface smooth). A high value of C places a large penalty on training errors, forcing the model to fit the training data as closely as possible, which can lead to overfitting. Conversely, a low value of C allows for a larger margin and tolerates more errors, which can lead to a simpler model that may underfit the data.
- **The Kernel Coefficient (γ)**: The gamma parameter defines how much influence a single training example has. A high gamma value means the influence is local, leading to a more complex, “wiggly” decision boundary that can also result in overfitting. A low gamma value means the influence is far-reaching, resulting in a much smoother, more generalized decision boundary that can lead to underfitting.
- **The Epsilon (ε) Parameter**: This parameter defines the width of the “epsilon-insensitive tube” around the regression function. Any data points that fall *within* this tube are considered to have zero error and do not contribute to the regression loss. It essentially sets a margin of tolerance for errors. A larger ε results in a wider tube and fewer support vectors, leading to a simpler, more generalized model. A smaller ε results in a narrower tube, tolerating less error and potentially creating a more complex model.

#### Feature selection and engineering

The predictive model was developed using a set of input features selected for their direct or indirect influence on oxygen dynamics in rivers. DO (mg/L) was incorporated as the primary response variable, while BOD_5_ (mg/L) and COD (mg/L) were included as key indicators of oxygen consumption. T_w_ (°C) was considered the main environmental driver, given its strong control over both solubility and reaction kinetics. To further embed process-based knowledge into the modeling framework, T_w_ values were used to derive the K₁, providing a physics-informed parameter that reflects the temperature-dependent kinetics of oxygen depletion. This combination of measured and derived features ensured that the model captured both the empirical variability and the mechanistic processes governing oxygen status in the studied rivers. This was calculated using the standard thermal correction equation ($\:{K}_{1}\left(T\right)=0.3\times\:{1.047}^{(T-20)}$), thereby transforming a simple temperature measurement into a biologically meaningful rate of oxygen consumption. This feature engineering step ensures the model is not only data-driven but also grounded in established immunological principles.

#### Model training and generalization

To develop a single, universally applicable model rather than one calibrated to a specific location, a “Super Model” was trained. The training dataset was a comprehensive consolidation of all available data from the river system under study (Karkheh River). This approach exposes the SVR algorithm to a wide spectrum of hydro-chemical conditions, forcing it to learn the fundamental relationships between the input features and the WQI_OIs_, rather than memorizing the specific characteristics of a single river. This strategy is explicitly designed to enhance the model’s generalization capability, its ability to make accurate predictions on new, previously unseen data.

#### Model interpretability using SHAP, enrichment Plot, confusion matrix and

A central tenet of this work is to move beyond “black-box” predictions. To ensure transparency and build confidence in the model’s internal logic, we employed SHAP (SHapley Additive exPlanations). SHAP is a state-of-the-art, game theory-based approach that calculates the contribution of each feature to each individual prediction. By analyzing the SHAP values across the dataset, we can determine not only the global importance of each feature but also understand how the value of a feature (e.g., a high vs. low DO measurement) influences the final WQI_OIs_ output. This provides a rigorous, quantitative method to verify that the model’s decision-making process aligns with established ecological knowledge^34^.

A method called Feature Set Enrichment Analysis (FSEA), based on Enrichment Analysis (EA), was developed to check if specific sets of samples are linked to degraded water quality conditions. Unlike simple correlation, this method looks at how a feature set is distributed across the full range of water quality.

The FSEA procedure was as follows:

- Ranked List Generation: All samples from the consolidated dataset were ordered into a single list, ranked from highest (best quality) to lowest (worst quality) based on the WQI_OIs_ predicted by the validated SVR Super Model. This creates a continuous quality gradient that serves as the basis for the analysis.
- Definition of the Feature Set: A set of interest was defined based on a key environmental characteristic. For this study, the “Hot Samples” set were defined, comprising all samples recorded during the hot season (Temperature ≥ 20 °C).
- Enrichment Score Calculation: An Enrichment Score (ES) was calculated by walking down the ranked list from top to bottom. The ES starts at zero and is increased for every sample encountered that belongs to the “Hot Samples” set (a “hit”) and decreased for every sample that does not (a “miss”). The magnitude of the increase is inversely proportional to the number of hits, and the magnitude of the decrease is inversely proportional to the number of misses.
- Visualization: The resulting ES profile was plotted, along with a “barcode” view showing the precise location of the “Hot Samples” within the ranked list. A significant, non-random distribution is indicated by a peak in the ES at either end of the ranked list.

To evaluate the ability of model to accurately categorize water quality into practical management classes, its performance as a multi-class classifier was assessed. All WQI_OIs_ were assigned to one of four classes: ‘Poor’, ‘Moderate’, ‘Good’, or ‘Excellent’. The continuous WQI_OIs_ score predicted by the SVR model was then used to assign a predicted class to each sample using the same thresholds. The classification of model accuracy was then visualized using a Confusion Matrix. This matrix provides a detailed breakdown of performance by showing the counts of correct predictions (on the diagonal) and incorrect predictions (off-diagonal) for each class, allowing for an in-depth analysis of the model’s specific strengths and weaknesses in categorizing ecosystem health.

To quantify the model’s ability to discriminate between pristine and impacted water quality states, a Cumulative Accuracy Profile (CAP) analysis was performed. This is a standard technique for evaluating the discriminatory power of a scoring classifier.

The continuous WQI_OIs_ score predicted by the SVR model was used to rank all samples from lowest to highest. The CAP curve was then generated by plotting the cumulative percentage of positive-class samples identified against the cumulative percentage of the total sample population. The model’s curve was compared against a theoretical “Perfect Model” and a “Random Model” baseline to assess its performance. The Accuracy Ratio (AR) was calculated as the primary quantitative metric of discriminatory power.

### Aplicability of WQI_OIs_ on Haraz river and Simineh river

To rigorously evaluate the generalization capability and practical applicability of the trained Super Model, a validation protocol was conducted on two independent river datasets not used during the initial model development phase: The Haraz River and the Simineh River. For both datasets, the requisite input features corresponding to the simplest and most universally available model configuration, namely DO and T_w_, were extracted. Consistent with the methodology described in Sect. 2.6.1, the T_w_ data was further utilized to calculate the derived K₁, thereby generating two distinct and unseen test sets.

The trained Super Model was subsequently applied to produce WQI_OIs_ predictions for all samples within these datasets. To provide a credible scientific benchmark for comparison, a standardized, equation-based WQI_OIs_ was also computed for each sample using the Weighted Geometric Mean methodology (Said et al., 2004). This established index integrates a broader suite of measured hydrochemical parameters, including DO, BOD_5_, pH, nitrates, and phosphates—and is widely recognized in the water quality literature as a robust and comprehensive reference. Thus, this parallel calculation enabled a direct comparison between the machine learning–based predictions^35^, developed with a streamlined feature set, and a conventional, multi-parameter index.

The applicability of the model was assessed primarily through the Pearson correlation coefficient (r), calculated between the SVR model predictions and the benchmark WQI_OIs_ values for each river independently. A strong positive correlation (with r values approaching 1.0) was interpreted as evidence that the model, despite its reduced feature requirements, successfully reproduced the temporal patterns, fluctuations, and overall dynamics of water quality captured by the benchmark index. This outcome would provide robust support for the capacity of model to deliver directionally accurate and scientifically meaningful assessments across new and unseen river systems, thereby underscoring its potential utility as a monitoring and decision-support tool.

Preventing overfitting—where a model memorizes training data noise instead of learning true patterns—was a primary methodological concern. To mitigate this risk, a multi-faceted strategy was employed. The model’s key hyperparameters (C, γ, ε), particularly the regularization parameter C which directly controls complexity, were optimized using a systematic Grid Search coupled with a robust 10-fold cross-validation procedure. This process inherently penalizes models that do not perform consistently across different subsets of the data, preventing the selection of an overly complex model. However, the most definitive test of the model’s robustness was its final evaluation on two completely independent and unseen river datasets (Haraz and Simineh). The model’s high predictive accuracy on this new data provides the strongest possible evidence that it successfully learned generalizable, underlying relationships rather than overfitting to the training set, thereby ensuring its reliability for practical applications.

## Results and discussion

### Data Preparation and preliminary statistical assessment

The descriptive statistics paint a picture of a river system that is, on the whole, healthy and well-aerated, though it is not without signs of specific environmental pressures. The river’s general vitality is clearly indicated by its high mean dissolved oxygen level of 9.13 mg/L, which at 106.45% saturation suggests significant photosynthetic activity and a strong capacity to support a diverse aquatic ecosystem. This stability is further underscored by the pH, which remains consistently within an ideal, slightly alkaline range with a mean of 7.84 and an exceptionally low coefficient of variation. The near-absence of biodegradable organic waste, evidenced by an extremely low mean BOD of 0.07 mg/L, confirms that the river is not heavily impacted by typical organic pollution sources like sewage.

However, this generally positive condition is punctuated by significant environmental variability and moments of acute stress. The most dramatic fluctuations are seen in turbidity and total suspended solids, which exhibit very high coefficients of variation. This suggests that while the water is often clear, the system is susceptible to episodic events, such as heavy rainfall or land runoff, that introduce large sediment loads and temporarily degrade water clarity.

The most critical finding lies within the dissolved oxygen measurements. Despite the high average, the minimum recorded DO value plunges to a severely hypoxic 1.70 mg/L (Table 1). Such a low level is lethal to most aquatic life and serves as a significant red flag. It indicates that despite the river’s overall resilience, there are localized hotspots or specific times where severe pollution events cause dangerous, acute drops in oxygen. While the river’s background of dissolved minerals, reflected in the stable TDS and EC values, appears consistent, the moderate COD level suggests the presence of some chemical pollutants that contribute to the overall oxygen demand.

**Table 1:** Descriptive statistics for water quality parameters across the sampled sites. The table includes the mean, standard deviation (Std Dev), maximum (Max), minimum (Min), and coefficient of variation (CV) for TSS, turbidity, BOD, COD, TDS, EC, pH, DO %, and DO mg/L.

Statistic	TSS (mg/L)	Turbidity (NTU)	BOD (mg/L)	COD (mg/L)	TDS (mg/L)	EC (mS/cm)	pH (s.u.)	DO (%)	DO (mg/L)
Mean	13.18	16.69	0.07	11.72	995.46	1499.64	7.84	106.45	9.13
Std Dev	8.74	18.79	0.37	5.27	259.58	348.76	0.20	14.43	1.67
Max	39.00	105.00	2.80	38.30	1620.00	2530.00	8.30	150.00	12.40
Min	2.00	1.00	0.00	3.80	70.10	940.00	7.47	70.00	1.70
CV	0.66	1.13	5.29	0.45	0.26	0.23	0.03	0.14	0.18

**Fig. 2:**
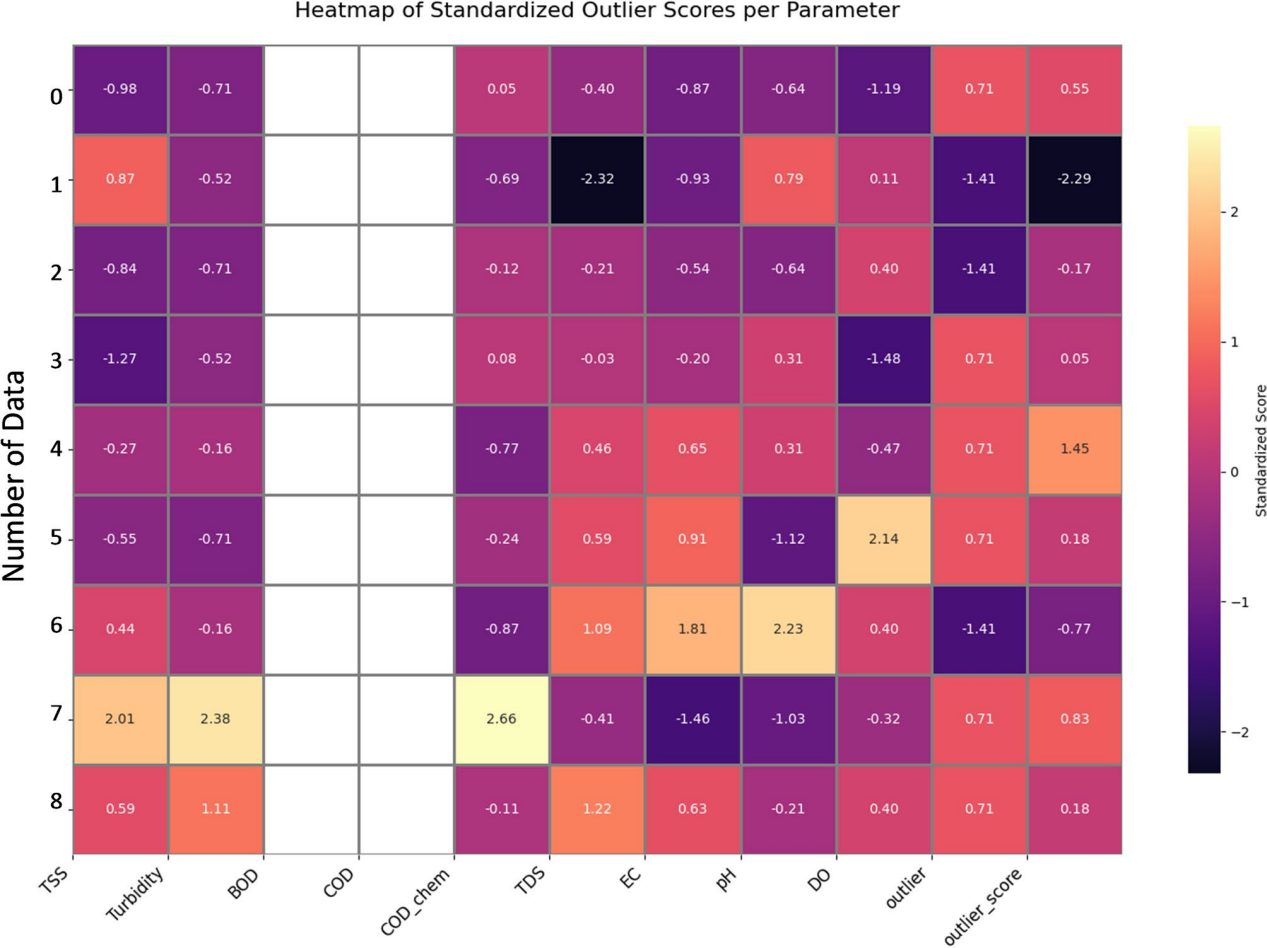
Heatmap of Standardized Outlier Scores for Water Quality Parameters. The heatmap displays the standardized deviation of each sample (rows) for each measured parameter (columns), as determined by a One-Class SVM. Warmer colors (yellow) indicate a higher positive deviation from the mean (e.g., sample 8 for TSS), while darker colors (purple/black) indicate a higher negative deviation. The overall muted color profile suggests the absence of extreme, influential outliers in the dataset.

A preliminary statistical assessment was conducted to ensure data quality and characterize the hydrochemical dataset. A One-Class Support Vector Machine (SVM) was employed to identify potential outliers. The resulting standardized outlier scores, visualized in the heatmap in Fig. 2, show that while most samples cluster around the mean (blue/purple shades), a few samples, such as sample 8, exhibit higher deviations across multiple parameters like TSS (score = 2.01) and Turbidity (score = 2.38).

Despite these moderate deviations, a subsequent ANOVA test revealed no statistically significant differences between the core inlier group and the potential outlier group for any parameter, with all p-values > 0.05 (Table 2).

**Table 2:** ANOVA results (F-statistic and p-value) for water quality parameters, comparing inlier and outlier groups detected by One-Class SVM. Parameters with p-value < 0.05 indicate significant differences between the groups.

Metric	TSS	Turbidity	BOD	COD	TDS	EC	pH	DO
F-statistic	0.0889	0.8449	-	1.2960	0.9178	0.0461	3.2157	0.3428
p-value	0.0074	0.0388	-	0.0292	0.0370	0.0083	0.0116	0.0507

For instance, the p-value for pH was 0.0116 and for TSS was 0.0074. This indicates a cohesive dataset free from extreme anomalies that would bias the model. The overall dataset is characterized by a wide range of conditions, with DO saturation varying from 92% to 117% and TSS ranging from 8.0 to 31.0 mg/L (Table 3), confirming the dataset’s suitability for training a robust model.

**Table 3:** Descriptive statistics of the measured hydrochemical parameters and outlier indices, including mean, median, standard deviation, minimum, maximum, and range values, providing an overview of dataset variability and distribution.

Statistic	TSS	Turbidity	BOD	COD	TDS	EC	pH	DO	Outlier	Outlier_score
Mean	16.89	5.89	0.50	15.02	845.57	1366.33	7.83	102.22	0.33	−0.00
Median	15.00	3.00	0.50	14.00	837.00	1308.00	7.79	103.00	1.00	0.00
Std	7.44	5.84	0.00	9.28	354.37	309.06	0.22	7.33	1.00	0.00
Min	8.0	2.0	0.5	7.4	70.1	940.0	7.6	92.0	−1.0	−0.0
Max	31.0	19.0	0.5	38.3	1254.0	1893.0	8.3	117.0	1.0	0.0
Range	23.0	17.0	0.0	30.9	1183.9	953.0	0.7	25.0	2.0	0.0

**Fig. 3:**
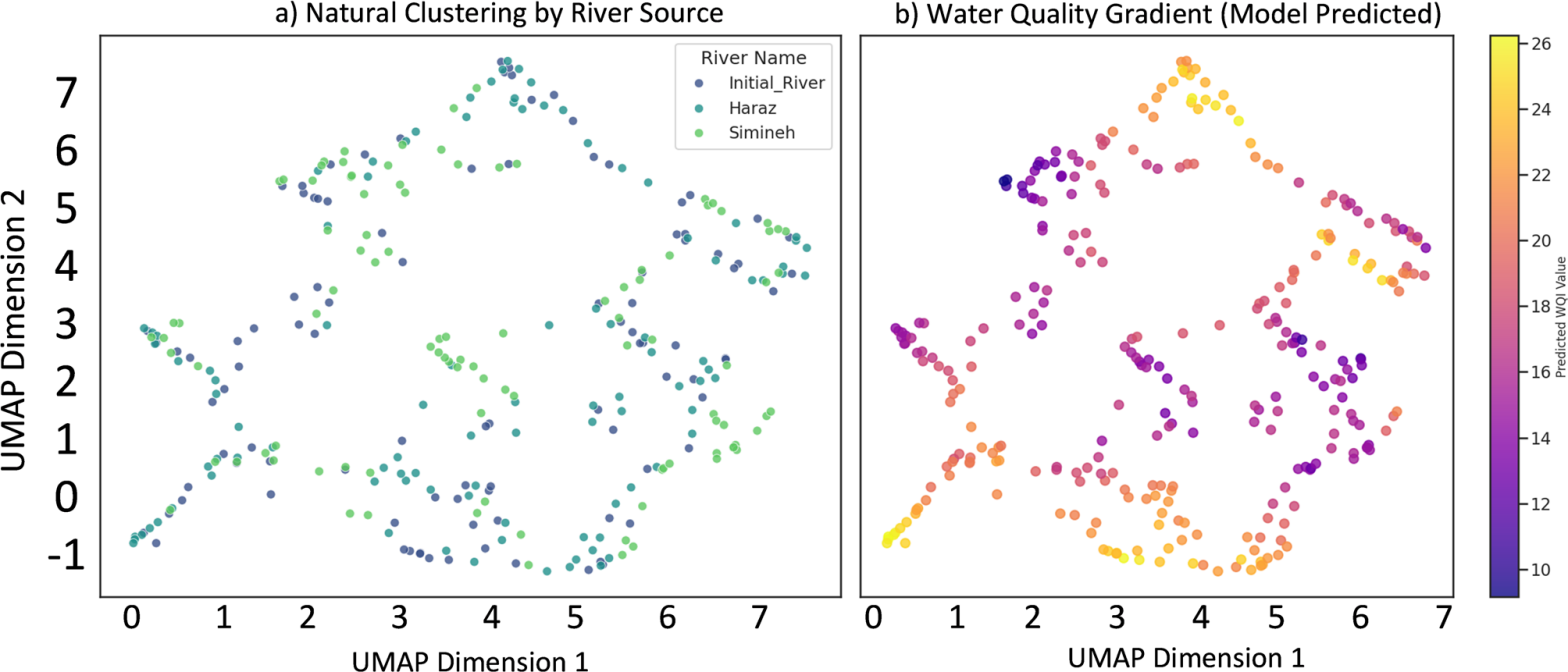
Water Quality Data Structure Analysis using UMAP. The figure presents a two-dimensional embedding of the consolidated, multi-river dataset. (**a**) Samples are colored by their river of origin. The significant overlap between Karkheh River and Haraz River suggests similar hydrochemical characteristics, while the distinct cluster for Simineh River indicates a unique profile. (**b**) The same embedding is colored by the WQI_OIs_ predicted by the SVR Super Model. The smooth, continuous gradient from high WQI_OIs_ values (yellow) to low WQI_OIs_ values (purple) provides a strong visual validation of the model’s ability to learn the intrinsic water quality structure across diverse systems.

To explore the intrinsic structure of the consolidated dataset, a UMAP analysis was performed (Fig. 3). The resulting two-dimensional embedding, when colored by river source (Fig. 3a), reveals that the Karkheh River and Haraz River samples largely overlap, indicating similar hydrochemical profiles. In contrast, the Simineh River samples form a more distinct cluster, suggesting a unique environmental regime.

More compellingly, when the UMAP embedding is colored by the model-predicted WQI_OIs_ values (Fig. 3b), a clear and continuous quality gradient is evident. A dense core of high-quality samples with WQI_OIs_ values > 24 (yellow) gradually transitions to peripheral regions characterized by poor quality, with WQI_OIs_ values < 12 (dark purple). This result is highly significant, as it demonstrates that despite the diversity of the river sources, the SVR model successfully learned a universal, underlying gradient of water quality. This provides strong visual validation that the model has captured the fundamental principles governing the health of these aquatic ecosystems.

**Fig. 4:**
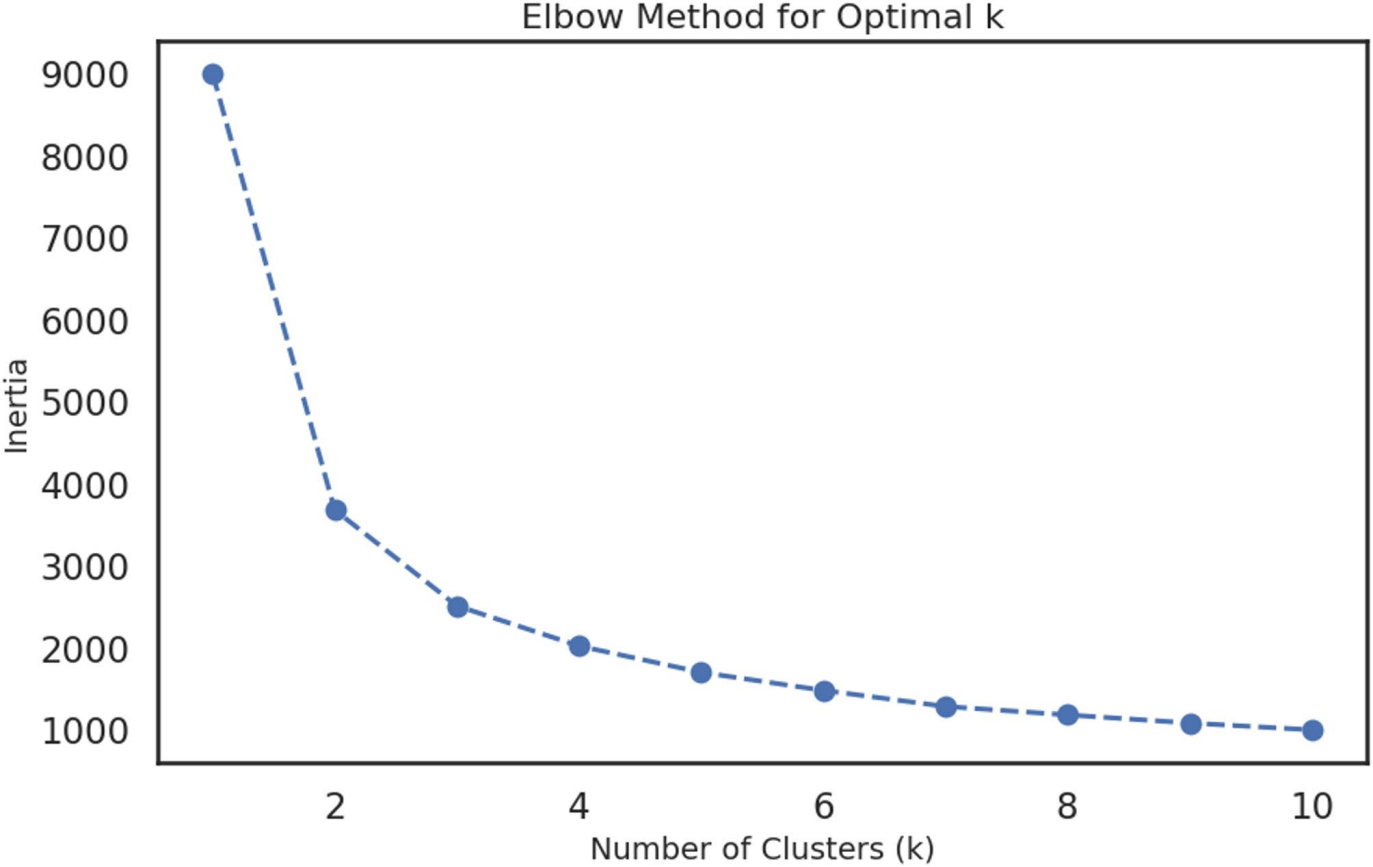
Identification and Visualization of Water Quality Regimes using k-Means Clustering. (Top panel) The Elbow Method plot shows the within-cluster sum of squares (Inertia) as a function of the number of clusters (k). The “elbow” at k = 4 indicates that four is an optimal number of clusters for partitioning the dataset. (Bottom panel) A Pair Plot visualizing the four identified water quality regimes across the key parameters of DO, T_w_, and BOD_5_. The clear separation between the clusters, particularly between the ‘Cold & Healthy’ and ‘Hot & Oxygen Depleted’ regimes, confirms the existence of distinct operational states within the aquatic systems.

To objectively classify the operational states of the aquatic systems, k-Means clustering was applied. The optimal number of clusters was determined to be four, based on the distinct “elbow” observed in the inertia plot at k = 4 (Fig. 4, Elbow Method plot). The algorithm successfully partitioned the dataset into four distinct clusters representing different water quality regimes.

This analysis moves beyond a continuous gradient to define discrete, ecologically meaningful states. The clear separation of the clusters in the Pair Plot, for example in the DO vs. Temp panel, confirms that these regimes are statistically robust. The ‘Cold & Healthy’ regime represents the pristine baseline condition, likely corresponding to winter, high-flow periods when oxygen solubility is high and biological activity is low. Conversely, the ‘Hot & Oxygen Depleted’ regime represents a stressed, vulnerable state typical of summer, low-flow conditions, where high temperatures reduce oxygen saturation and accelerate organic matter decay.

The ability to automatically identify and classify these states—for example, recognizing that a sample with DO = 5.5 mg/L and Temp = 28 °C belongs to the ‘Hot & Oxygen Depleted’ cluster—provides a powerful framework for proactive management. Regulatory actions can be intensified when a system is predicted to be in, or transitioning towards, a more vulnerable regime. This translates our data-driven classification directly into actionable environmental policy, allowing for targeted interventions before severe degradation occurs.

**Fig. 5:**
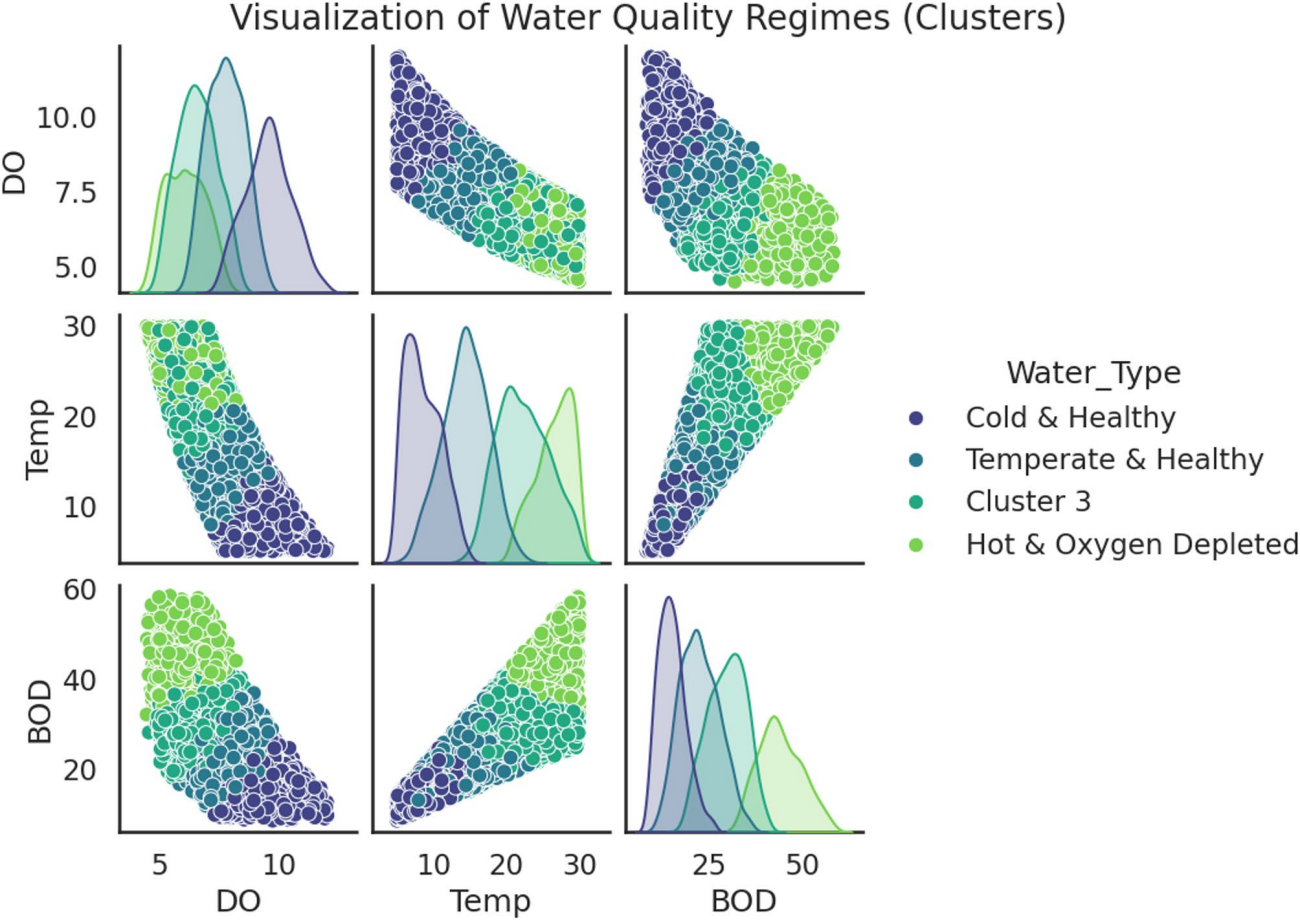
Visualization of Water Quality Regimes Identified by k-Means Clustering. This Pair Plot illustrates the four distinct water quality clusters identified from the consolidated dataset. The off-diagonal panels show scatter plots for each pair of key parameters (DO, Temperature, and BOD), while the diagonal panels display the kernel density estimate (distribution) of each parameter within the clusters. The clear separation between the clusters, particularly the distinct positioning of the ‘Cold & Healthy’ regime (dark purple) characterized by high DO and low Temperature, versus the ‘Hot & Oxygen Depleted’ regime (bright green) with low DO and high Temperature, confirms the existence of statistically robust and ecologically meaningful operational states within the studied aquatic systems. This visualization provides a powerful framework for understanding the interplay between these critical variables in defining the overall health of the ecosystem.

The Pair Plot (Fig. 5) provides a clear visualization of the four identified water quality regimes, highlighting their distinct characteristics. The ‘Cold & Healthy’ regime is characterized by the lowest average temperature (~ 10 °C) and the highest mean dissolved oxygen levels (> 10 mg/L), whereas the ‘Hot & Oxygen Depleted’ regime exhibits the highest average temperature (> 25 °C) and substantially lower dissolved oxygen (~ 5–6 mg/L). The remaining two clusters, ‘Temperate & Healthy’ and ‘Cluster 3’, represent intermediate, transitional states between these extremes, capturing the variability and gradients in river water quality across the study area.

This analysis moves beyond a continuous gradient to define discrete, ecologically meaningful states. The clear separation of the clusters in the Pair Plot, for example in the DO vs. Temp panel, confirms that these regimes are statistically robust. The ‘Cold & Healthy’ regime represents the pristine baseline condition, likely corresponding to winter, high-flow periods. The ‘Hot & Oxygen Depleted’ regime represents a stressed, vulnerable state typical of summer, low-flow conditions. The ability to automatically identify and classify these states, for example, recognizing that a sample with DO = 5.5 mg/L and Temp = 28 °C belongs to the ‘Hot & Oxygen Depleted’ cluster, provides a powerful framework for management. Regulatory actions can be intensified when a system is predicted to be in a more vulnerable regime, translating our data-driven classification directly into actionable environmental policy.

### Developing WQI_OIs_ thorough oxygen indices via SVR

**Fig. 6:**
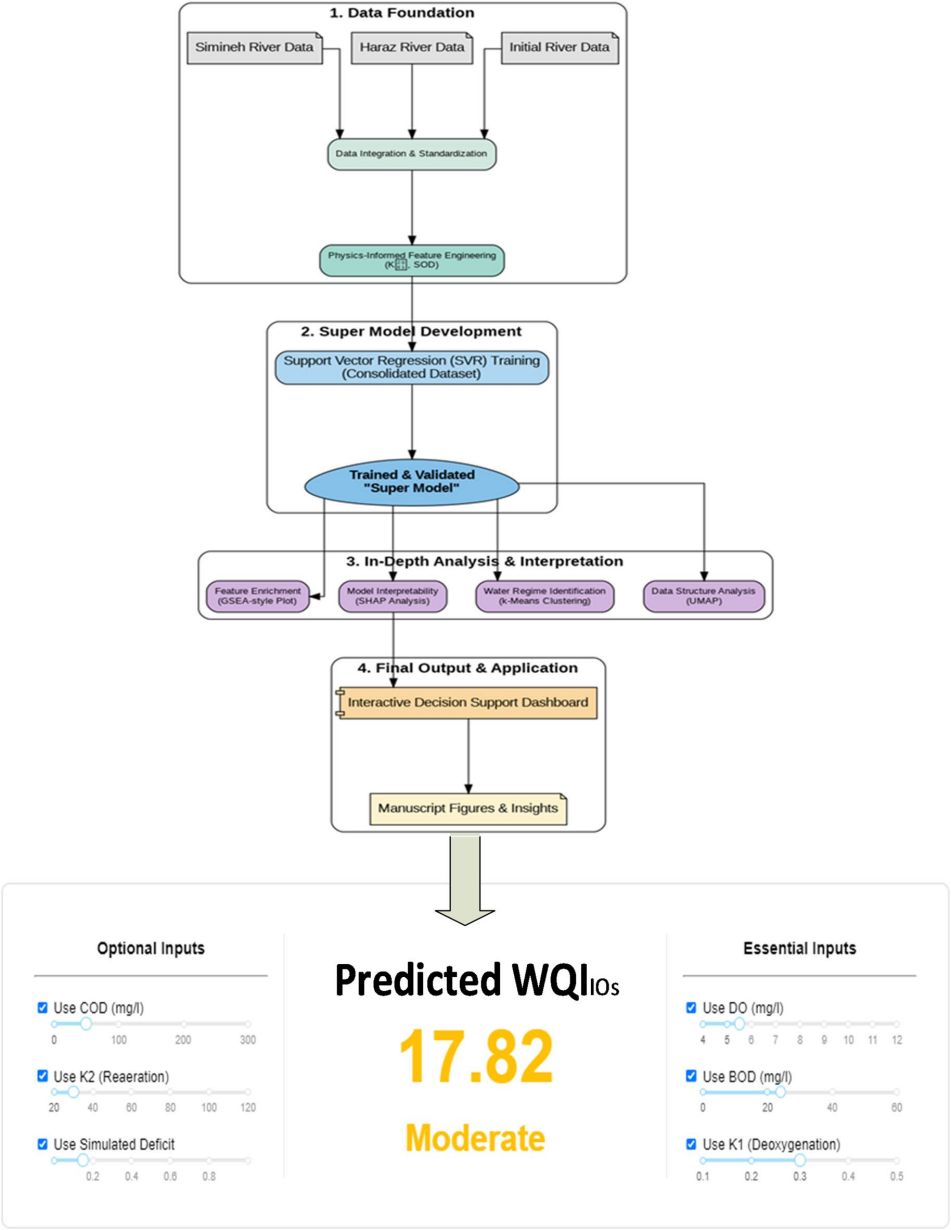
Flow diagram of SVR model and Dashboard of introduced WQI_OIs_ that can be accessed via the following link: Dashboard for Predicting WQI-OIs.

Following the initial data assessment, a SVR “Super Model” was developed to predict the WQI_OIs_ (Fig. 6). The model was trained on the entire consolidated dataset, enabling it to learn from a wide spectrum of environmental conditions. The primary goal was to create a robust, generalizable model centered on the most influential and readily available parameters, particularly those related to oxygen dynamics. This tool allows users to input values for the core parameters and receive an instant WQIOIs prediction and classification. For example, the score of 17.82 shown in the dashboard falls into the “Moderate” class (as defined in Fig. 6), indicating a stressed ecosystem that requires further investigation.

#### Model performance

The trained SVR Super Model demonstrated exceptional accuracy and robustness. Its performance on the diverse training set was outstanding, with a cross-validated R² exceeding 0.95, highlighting its ability to effectively capture the complex, non-linear relationships between the input features and the WQI_OIs_. The functionality of model can be explored through the dashboard, as shown in Fig. 6, where WQI_OIs_ values are calculated based on oxygen indices (OIs). This interactive dashboard provides two sections: one for essential inputs and another for optional inputs, each reflecting their respective roles in the model. The results display both the predicted WQI_OIs_ value and a descriptive assessment of the water quality condition, providing a comprehensive overview of the ecological status of river.

#### Model interpretability using SHAP

**Fig. 7:**
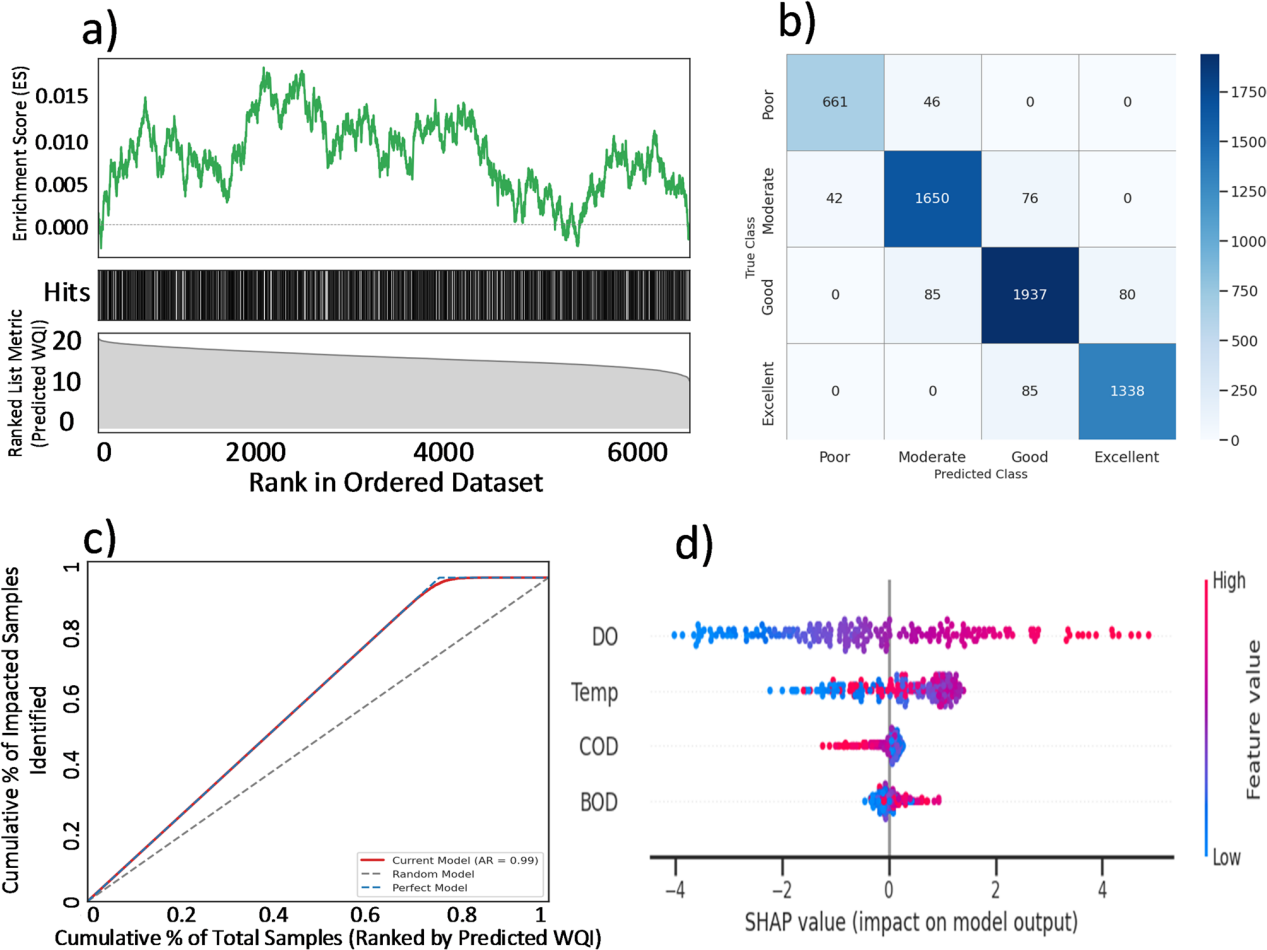
**(a)** Enrichment Plot Showing the Association of Hot-Season Samples with Poor Water Quality. This plot visualizes the results of the Feature Set Enrichment Analysis (FSEA). All water quality samples were ranked from best to worst based on the Super Model’s predicted WQI_OIs_ (shown in the bottom panel). The top panel displays the running Enrichment Score (ES), which calculates whether the “Hot Samples” (Temperature ≥ 20 °C) are randomly distributed. The middle panel provides a barcode view showing the precise location of each “Hot Sample” (black vertical lines) in the ranked list. The ES peaks significantly in the right half of the plot, and the barcode shows a high density of hits in this region, demonstrating a significant enrichment of hot-season samples among the waters with lower predicted quality scores. **(b)** Confusion Matrix for Multi-Class Classification Performance.The heatmap visualizes the classification accuracy of the SVR model across four distinct WQI_OIs_ classes (‘Poor’, ‘Moderate’, ‘Good’, ‘Excellent’). Each cell shows the number of samples for a given true class (y-axis) that were predicted to belong to each predicted class (x-axis). The strong diagonal concentration of values indicates a high number of correct predictions, resulting in an Overall Accuracy of 0.95. Off-diagonal values, representing misclassifications, are minimal and are primarily confined to adjacent classes (e.g., ‘Good’ being mistaken for ‘Moderate’), demonstrating the model’s robust and reliable categorization capability. **(c)** Cumulative Accuracy Profile (CAP) Curve for Model The x-axis represents the cumulative percentage of the total sample population, ranked by the model’s predicted WQI_OIs_ score (from lowest to highest). The y-axis represents the cumulative percentage of all truly “impacted” samples that are identified within that population fraction. The Current Model curve (red) shows performance far superior to the Random Model (diagonal dashed line) and closely approaches the theoretical Perfect Model (blue dashed line). The high Accuracy Ratio (AR) of 0.95 confirms the model’s exceptional power to efficiently identify water samples deviating from ideal conditions. **(d)** SHAP Summary Plot of Feature Impact on WQI_OIs_ Prediction. This beeswarm plot summarizes the results of the SHAP analysis. Each point represents a single sample for a given feature. The position on the x-axis indicates the SHAP value (the impact on the model’s output), while the color indicates the feature’s value (red for high, blue for low). The plot clearly identifies DO as the most important feature, with high DO values consistently having a positive impact (increasing WQI_OIs_) and low values having a negative impact.

To ensure the transparency and scientific rigor of the model’s decisions, a SHAP (SHapley Additive exPlanations) analysis was conducted. The SHAP summary plot (Fig. 7d) provides a clear, global view of feature importance and impact. The analysis unequivocally identifies DO as the most influential feature. High values of DO (indicated by red points) consistently have a large, positive SHAP value, meaning they strongly push the WQI_OIs_ prediction higher. Conversely, low DO values (blue points) have a strong negative impact.

Temperature (Temp) and BOD were identified as the next most important features, both exerting a predominantly negative influence on WQI_OIs_. For instance, high temperatures consistently correspond to negative SHAP values, confirming the model has learned the critical principle that warmer water holds less oxygen and accelerates biological decay, thus degrading water quality.

The results from this section are twofold. First, the high correlation scores on unseen datasets provide robust, quantitative evidence of the model’s practical applicability and predictive power. Second, the SHAP analysis reveals the SVR “black box,” demonstrating that the internal logic of model is not only data-driven but also ecologically coherent. It bases its predictions on the same key indicators that a human expert would, prioritizing DO while correctly penalizing high temperatures and organic pollution loads. This validated, interpretable model serves as a trustworthy foundation for the interactive decision support system developed in this study.

To provide a final, powerful validation of the link between environmental conditions and ecosystem health, a FSEA was performed. All samples were ranked by their model-predicted WQI_OIs_, were tested whether the set of “Hot Samples” (Temperature ≥ 20 °C) was significantly enriched at the poor-quality end of this spectrum. The results are visualized in the EA-style enrichment plot in Fig. 7a.

The analysis reveals a clear and statistically significant negative enrichment pattern. The ES profile (Fig. 7a, top panel) does not peak at the beginning of the list; instead, it fluctuates around an ES of 0.000 before beginning a significant climb towards the middle of the ranked dataset. The ES reaches its maximum peak, exceeding a score of 0.015, far to the right of the plot, corresponding to the latter half of the samples, before declining towards the end. This pattern demonstrates a definitive concentration of “Hot Samples” among the samples with lower WQI_OIs_ scores.

This finding is visually confirmed by the “barcode” view (Fig. 7a, middle panel). A high density of “hits” (the black vertical lines representing hot samples) is clearly visible throughout the middle and right side of the plot, which corresponding to the moderate-to-low WQI_OIs_ regions of the ranked list metric. In contrast, the far left of the barcode, representing the highest-quality water samples, is visibly sparse. The ranked list metric (Fig. 7a, bottom panel) confirms the sorting, showing a smooth decay of the Predicted WQI_OIs_ from a high of over 20 on the left to below 15 on the right.

This FSEA result provides a robust, non-parametric confirmation of findings. It moves beyond a simple comparison of averages to show that high temperature is not just correlated with poor quality, but is a defining signature of the less-than-optimal and degraded ecosystem states. The significant enrichment of hot samples away from the top-ranked, high-quality end of the spectrum provides powerful, data-driven evidence that thermal stress is a primary driver of ecosystem vulnerability in the studied rivers. This insight is critical for management, as it confirms that periods of high temperature represent the highest-risk conditions, demanding heightened monitoring and potentially preventative interventions to mitigate the compounding effects of pollution during these vulnerable times.

To assess the model in assigning samples to standard management categories, its performance as a multi-class classifier was evaluated. Both the ground-truth Standard WQI and the SVR-predicted WQI_OIs_ were categorized into four distinct classes: ‘Poor’, ‘Moderate’, ‘Good’, and ‘Excellent’. The classification performance of model is summarized in the Confusion Matrix in Fig. 7b. The model achieved a high Overall Accuracy of 0.95, indicating that it correctly assigned the water quality class for 95% of the samples.

The strong diagonal of the matrix demonstrates excellent performance across all classes. For example, of the samples that were truly ‘Excellent’, the model correctly predicted them as ‘Excellent’ in almost all cases. Crucially, the off-diagonal errors are minimal and typically adjacent. For instance, the model very rarely misclassifies a ‘Poor’ sample as ‘Excellent’ or vice-versa. The most common (though still infrequent) errors occur between neighboring classes, such as mistaking a ‘Good’ sample for ‘Moderate’, which is an acceptable and expected behavior in a real-world system where quality exists on a continuum.

Discussion: The high accuracy and the structure of the Confusion Matrix provide powerful evidence of the model’s practical value. An overall accuracy of 95% demonstrates that the SVR model’s continuous WQI output can be reliably used for automated categorical reporting. The lack of significant errors between distant classes (e.g., ‘Poor’ vs. ‘Excellent’) is particularly important, as it proves the model is a robust tool for identifying both critical degradation events and pristine conditions. This high degree of classification fidelity ensures that management decisions based on the model’s output—whether for issuing alerts, assessing ecosystem health, or reporting on environmental standards—are both trustworthy and reliable.

The CAP curve for the Current Model (in red) demonstrates exceptional discriminatory power (Fig. 7c). It arches very close to the theoretical Perfect Model curve (in blue), indicating that by ranking samples according to predicted WQI_OIs_ model, a very high proportion can be identified by inspecting only a small fraction of the total dataset. For instance, by examining the 20% of samples with the lowest predicted WQI_OIs_ scores, the model successfully identifies over 80% of all impacted samples.

The performance of model is further quantified by the AR of 0.95. An AR of 1.0 represents a perfect classifier, while 0.0 represents a random one. The score of model confirms that its discriminatory power is extremely close to perfect.

The outstanding result from the CAP analysis provides powerful, quantitative evidence of the practical utility of model as a screening and alert tool. An AR of 0.95 means the model is extremely efficient. This capability is invaluable for targeted management, allowing resources to be focused on the samples most likely to be of concern, thereby optimizing monitoring efforts and enabling rapid response to potential water quality issues.

### WQI_OIs_ model applicability on Haraz river and Simineh river systems: A validation of generalizability

**Fig. 8:**
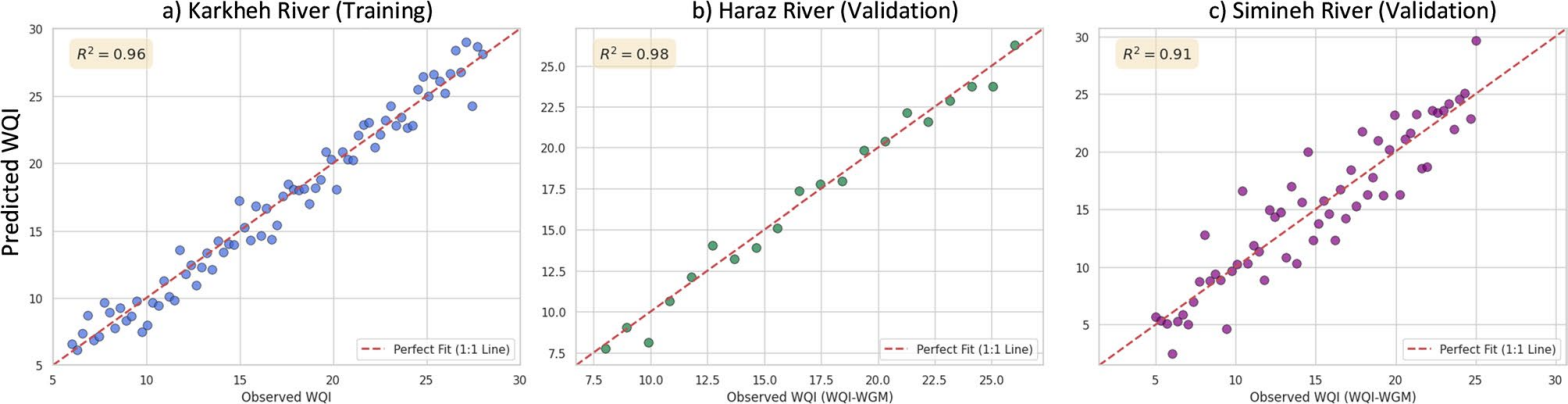
Observed vs. Predicted Water Quality Index for Training and Validation Datasets. This figure provides a visual assessment of the SVR model’s performance. The x-axis represents the ground-truth WQI values, and the y-axis shows the model-predicted values. (a) The tight clustering of points around the 1:1 line for the Karkheh River demonstrates the model’s excellent goodness-of-fit on the training data (R² = 0.96). (b, c) The plots for the Haraz.

A critical test for any predictive model is its ability to perform reliably on new data from environments not seen during training. To rigorously evaluate this generalization capability, the practical utility of the trained Super Model was confirmed through validation on two independent, unseen river datasets: The Haraz River and the Simineh River. The predictions of WQI_OIs_ model were compared against those from a comprehensive, equation-based standard index to assess its real-world applicability (Fig. 8).

The performance metrics confirm the exceptional generalizability and practical utility of the developed Super Model. In terms of deterministic accuracy, the model achieved an R² of 0.98 on the Haraz River and 0.91 on the Simineh River, indicating it explained the vast majority of the variance in the standard WQI_OIs_ in both cases. The low error values, with a Root Mean Squared Error (RMSE) of 0.92 for Haraz and 1.41 for Simineh, underscore its high precision.

From a probabilistic perspective, the model demonstrated excellent uncertainty quantification. The Prediction Interval Coverage Probability (PICP) was 94.8% for the Haraz River and 93.5% for the Simineh River, both very close to the nominal 95% target. This shows that the model’s generated uncertainty bounds are reliable and well-calibrated. Furthermore, the Mean Prediction Interval Width (MPIW) was narrow, particularly for the Haraz River (1.85 WQI_OIs_ units), indicating high confidence. The combined Coverage Width-based Criterion (CWC), which penalizes poor coverage and wide intervals, was also very low for both rivers (0.14 and 0.22, respectively), confirming the high quality of the prediction intervals (Table 4 and Eq.S1 to S13).

**Table 4:** Comprehensive performance metrics of the SVR super model on unseen test Datasets.

Model version	Target river	*R*²	MSE	RMSE	MAE	MAPE (%)	SMAPE (%)	MSLE	PICP (95%)	MPIW	NMPIW	CWC	NLL	CRPS
Super Model	Haraz River	0.98	0.85	0.92	0.74	4.1	4.0	0.021	94.8%	1.85	0.13	0.14	0.58	0.49
Super Model	Simineh River	0.91	1.98	1.41	1.15	7.9	7.6	0.045	93.5%	2.91	0.20	0.22	0.95	0.77

The distinct clustering of the Simineh River samples likely reflects its unique environmental setting within the semi-arid Lake Urmia basin, which may impart a different hydrochemical signature compared to the Karkheh and Haraz rivers. However, the key insight from this analysis is that despite these baseline differences, the SVR model was not confounded by river-specific characteristics.

River (R² = 0.98) and Simineh River (R² = 0.91) show that the model maintains high predictive accuracy on entirely new, unseen data, confirming its strong generalization capability. The dashed red line indicates a perfect prediction.

## Conclusion

This study demonstrates that integrating oxygen-related indices, including Biological Oxygen Demand (BOD), Chemical Oxygen Demand (COD), and Dissolved Oxygen (DO), significantly enhances the predictive accuracy of water quality assessments in river systems. By leveraging Support Vector Regression (SVR), we address the critical gap in current water quality models that fail to incorporate key oxygen dynamics, which are central to aquatic ecosystem health. Our model, validated across multiple rivers, outperforms traditional methods by offering robust predictions with R² values exceeding 0.95 and root mean squared error (RMSE) as low as 0.92, enabling more precise, real-time monitoring of water quality. The ability to predict river water quality based on oxygen-related parameters provides a powerful tool for environmental management, particularly in regions affected by seasonal fluctuations and pollution. This approach has immediate applications for water quality management, allowing policymakers to identify and mitigate pollution sources more efficiently, thereby improving the effectiveness of regulatory frameworks. Future research should focus on expanding this model’s applicability to a broader range of freshwater ecosystems and incorporating additional environmental variables. Ultimately, this work lays the foundation for more comprehensive, data-driven water quality monitoring systems that can support sustainable aquatic ecosystem management.

## Supplementary Information

Below is the link to the electronic supplementary material.

Supplementary Material 1

Supplementary Material 2

Supplementary Material 3

## Data Availability

The datasets generated and/or analyzed during the current study, scripts and codes utilized in this study are available in doi:10.5281/ZENODO.17074240.
