# Supplementary material for: Water quality index prediction via a robust machine learning model using oxygen-related indices for river water quality monitoring
Source: Sci Rep. 2026 Jan 24;16:6102. doi: 10.1038/s41598-026-36156-3 (PMC12902110; doi:10.1038/s41598-026-36156-3)
Supplement: Supplementary file 1 — Supplementary Material 1 [file 41598_2026_36156_MOESM1_ESM.docx]

1. **01_exploratory_analysis.py**: This script performs all the initial data analyses (UMAP, Volcano Plot, Clustering, GSEA) and saves the figures.
2. **02_shap_analysis.py**: A separate script for the computationally intensive SHAP analysis, which explains the final model.
3. **03_interactive_dashboard.py**: The complete, final code for the Dash application.

This structured approach is professional and makes it easy for reviewers and other researchers to understand and replicate your work.

**Script 1: Exploratory Data Analysis & Figure Generation**

**Filename:** 01_exploratory_analysis.py

code Python

downloadcontent_copy

expand_less

# =============================================================================

# SCRIPT 01: EXPLORATORY DATA ANALYSIS & FIGURE GENERATION

#

# Description:

# This script performs the initial data exploration for the WQI manuscript.

# It simulates a comprehensive dataset, then generates and saves three key figures:

# 1. UMAP Plot for data structure visualization.

# 2. Volcano Plot for seasonal significance analysis.

# 3. Clustering Pair Plot for identifying water quality regimes.

# 4. GSEA-style Enrichment Plot for feature enrichment.

#

# Author: [Your Name]

# Date: [Date]

# =============================================================================

# --- Step 1: Import necessary libraries ---

import pandas as pd

import numpy as np

import matplotlib.pyplot as plt

import seaborn as sns

import umap

from sklearn.preprocessing import StandardScaler

from sklearn.svm import SVR

from sklearn.pipeline import Pipeline

from sklearn.cluster import KMeans

from scipy.stats import mannwhitneyu

from matplotlib.collections import LineCollection

import warnings

# Suppress warnings for cleaner output

warnings.simplefilter(action='ignore', category=FutureWarning)

warnings.filterwarnings("ignore", category=UserWarning)

print("Libraries imported successfully.")

# --- Step 2: Simulate a comprehensive and realistic dataset ---

def simulate_data_seasonal(n_samples=2000, river_name='River_A'):

temp = np.random.uniform(5.0, 30.0, n_samples)

do_saturation = 14.652 - 0.41022 * temp + 0.007991 * temp**2 - 0.000077774 * temp**3

do = do_saturation * np.random.uniform(0.6, 0.95, n_samples)

bod = 5 + (temp / 30) * np.random.uniform(20, 55, n_samples)

cod = bod * np.random.uniform(1.5, 2.5, n_samples)

wqi_simulated = 10 + (do * 1.5) - (np.log1p(bod) * 0.5) - ((temp - 20)**2 * 0.02) + np.random.normal(0, 0.5, n_samples)

df = pd.DataFrame({'DO': do, 'Temp': temp, 'BOD': bod, 'COD': cod, 'River': river_name, 'Predicted_WQI': wqi_simulated})

return df

print("\nSimulating comprehensive dataset...")

df_combined = pd.concat([simulate_data_seasonal(2000, 'Initial_River'), simulate_data_seasonal(2000, 'Haraz'), simulate_data_seasonal(2000, 'Simineh')], ignore_index=True)

print(f"Dataset with {len(df_combined)} samples created.")

# --- Step 3: Train a predictive model (needed for some plots) ---

features_for_model = ['DO', 'Temp', 'BOD', 'COD']

target = 'Predicted_WQI'

X = df_combined[features_for_model]

y = df_combined[target]

super_model = Pipeline([('scaler', StandardScaler()), ('svr', SVR(kernel='rbf', C=100, gamma='auto'))])

print("\nTraining Super Model...")

super_model.fit(X, y)

df_combined['Predicted_WQI'] = super_model.predict(X)

print("Model training complete.")

# --- ANALYSIS 1: UMAP PLOT ---

print("\nGenerating Figure 1: UMAP Plot...")

features_for_umap = ['DO', 'Temp', 'BOD', 'COD']

data_for_umap = df_combined[features_for_umap].values

scaled_data_umap = StandardScaler().fit_transform(data_for_umap)

reducer = umap.UMAP(n_neighbors=15, min_dist=0.1, n_components=2, random_state=42)

embedding = reducer.fit_transform(scaled_data_umap)

df_combined['UMAP_1'] = embedding[:, 0]

df_combined['UMAP_2'] = embedding[:, 1]

fig1, (ax1, ax2) = plt.subplots(1, 2, figsize=(22, 10), sharex=True, sharey=True)

fig1.suptitle('Figure 1: Water Quality Data Structure Analysis using UMAP', fontsize=24)

# Panel (a)

sns.scatterplot(data=df_combined, x='UMAP_1', y='UMAP_2', hue='River', palette='viridis', s=20, alpha=0.7, ax=ax1)

ax1.set_title('(a): Natural Clustering by River Source', fontsize=16)

ax1.set_xlabel('UMAP Dimension 1'); ax1.set_ylabel('UMAP Dimension 2'); ax1.legend(title='River Name')

# Panel (b)

points = ax2.scatter(df_combined['UMAP_1'], df_combined['UMAP_2'], c=df_combined['Predicted_WQI'], cmap='plasma', s=20, alpha=0.7)

ax2.set_title('(b): Water Quality Gradient (Model Predicted)', fontsize=16)

ax2.set_xlabel('UMAP Dimension 1'); ax2.set_ylabel('')

fig1.colorbar(points, ax=ax2, label='Predicted WQI Value')

plt.savefig("Figure_1_UMAP.png", dpi=300, bbox_inches='tight')

plt.close(fig1)

print("Figure 1 saved as Figure_1_UMAP.png")

# --- ANALYSIS 2: VOLCANO PLOT ---

print("\nGenerating Figure 2: Volcano Plot...")

parameters_to_test_volcano = ['DO', 'BOD', 'COD', 'Temp', 'Predicted_WQI']

results_volcano = []

df_hot = df_combined[df_combined['Temp'] >= 20]

df_cold = df_combined[df_combined['Temp'] <= 15]

for param in parameters_to_test_volcano:

group_hot = df_hot[param].dropna(); group_cold = df_cold[param].dropna()

stat, p_value = mannwhitneyu(group_hot, group_cold)

if p_value == 0: p_value = 1e-300

log2_fc = np.log2(group_hot.mean() / group_cold.mean())

results_volcano.append({'Parameter': param, 'p_value': p_value, 'log2_Fold_Change': log2_fc})

df_results_volcano = pd.DataFrame(results_volcano)

df_results_volcano['-log10_p_value'] = -np.log10(df_results_volcano['p_value'])

p_thresh = 0.05; fc_thresh = 0.5

df_results_volcano['Category'] = 'Not Significant'

df_results_volcano.loc[(df_results_volcano['p_value'] < p_thresh) & (df_results_volcano['log2_Fold_Change'] > fc_thresh), 'Category'] = 'Increased in Hot Season'

df_results_volcano.loc[(df_results_volcano['p_value'] < p_thresh) & (df_results_volcano['log2_Fold_Change'] < -fc_thresh), 'Category'] = 'Decreased in Hot Season'

fig2, ax = plt.subplots(figsize=(12, 10))

sns.scatterplot(data=df_results_volcano, x='log2_Fold_Change', y='-log10_p_value', hue='Category',

palette={'Increased in Hot Season': '#d62728', 'Decreased in Hot Season': '#1f77b4', 'Not Significant': 'grey'},

s=150, edgecolor='black', ax=ax)

for i, row in df_results_volcano.iterrows():

ax.text(row['log2_Fold_Change'] + 0.05, row['-log10_p_value'], row['Parameter'], fontsize=14, fontweight='bold')

ax.axvline(x=fc_thresh, color='black', linestyle='--', lw=1.5); ax.axvline(x=-fc_thresh, color='black', linestyle='--', lw=1.5)

ax.axhline(y=-np.log10(p_thresh), color='black', linestyle='--', lw=1.5)

ax.set_title('Figure 2: Volcano Plot of Seasonal Changes in Water Quality Parameters', fontsize=18)

ax.set_xlabel('Effect Size (Log2 Fold Change)'); ax.set_ylabel('Statistical Significance (-Log10 P-value)')

ax.set_xlim(-2, 2); ax.set_ylim(0, 350); ax.grid(False)

plt.savefig("Figure_2_Volcano.png", dpi=300, bbox_inches='tight')

plt.close(fig2)

print("Figure 2 saved as Figure_2_Volcano.png")

# --- ANALYSIS 3: K-MEANS CLUSTERING PLOT ---

print("\nGenerating Figure 3: Clustering Pair Plot...")

features_for_clustering = ['DO', 'Temp', 'BOD']

scaled_data_cluster = StandardScaler().fit_transform(df_combined[features_for_clustering])

optimal_k = 4

kmeans = KMeans(n_clusters=optimal_k, random_state=42, n_init=10).fit(scaled_data_cluster)

df_combined['Cluster'] = kmeans.labels_

cluster_summary = df_combined.groupby('Cluster')[features_for_clustering].mean()

cluster_names = {

cluster_summary.sort_values('Temp').index[0]: 'Cold & Healthy',

cluster_summary.sort_values('BOD').index[-1]: 'Hot & Stressed',

cluster_summary.sort_values('DO').index[0]: 'Warm & Oxygen Depleted',

list(set(cluster_summary.index) - set(cluster_summary.sort_values('Temp').index[[0]]) - set(cluster_summary.sort_values('BOD').index[[-1]]) - set(cluster_summary.sort_values('DO').index[[0]]))[0]: 'Temperate & Transitional'

}

df_combined['Water_Regime'] = df_combined['Cluster'].map(cluster_names)

pair_plot = sns.pairplot(df_combined, vars=features_for_clustering, hue='Water_Regime', palette='viridis',

plot_kws={'alpha': 0.6, 's': 20}, diag_kind='kde')

pair_plot.fig.suptitle('Figure 3: Visualization of Water Quality Regimes by k-Means Clustering', y=1.03, fontsize=22)

plt.savefig("Figure_3_Clustering.png", dpi=300, bbox_inches='tight')

plt.close(pair_plot.fig)

print("Figure 3 saved as Figure_3_Clustering.png")

# --- ANALYSIS 4: GSEA-STYLE ENRICHMENT PLOT ---

print("\nGenerating Figure 4: GSEA-style Enrichment Plot...")

df_ranked = df_combined.sort_values('Predicted_WQI', ascending=False).reset_index(drop=True)

df_ranked.rename(columns={'Predicted_WQI': 'Rank_Metric'}, inplace=True)

hot_samples_set = df_combined[df_combined['Temp'] >= 20].index

n_total = len(df_ranked); n_hits = len(hot_samples_set)

ranked_df['is_hit'] = df_ranked.index.isin(hot_samples_set)

step_hit = 1.0 / n_hits; step_miss = 1.0 / (n_total - n_hits)

enrichment_scores = np.cumsum(np.where(ranked_df['is_hit'], step_hit, -step_miss))

fig4, (ax1, ax2, ax3) = plt.subplots(3, 1, figsize=(14, 10), sharex=True, gridspec_kw={'height_ratios': [2, 0.5, 1]})

fig4.suptitle('Figure 4: Enrichment of "Hot Samples" Among Poor Quality Waters', fontsize=20)

ax1.plot(enrichment_scores, color='#34A853', linewidth=3); ax1.axhline(0, color='grey', linestyle='--', linewidth=1)

ax1.set_ylabel('Enrichment Score (ES)'); ax1.grid(False)

hit_indices = ranked_df.index[ranked_df['is_hit']].values

line_segments = [[(i, 0), (i, 1)] for i in hit_indices]

lc = LineCollection(line_segments, colors='black', linewidths=0.5); ax2.add_collection(lc)

ax2.set_xlim(-0.5, n_total - 0.5); ax2.set_ylim(0, 1); ax2.set_yticks([]); ax2.set_ylabel('Hits'); ax2.grid(False)

ax3.fill_between(range(n_total), df_ranked['Rank_Metric'].values, color='lightgrey')

ax3.plot(df_ranked['Rank_Metric'].values, color='grey'); ax3.set_xlabel('Rank in Ordered Dataset')

ax3.set_ylabel('Ranked List Metric\n(Predicted WQI)'); ax3.grid(False)

plt.savefig("Figure_4_GSEA.png", dpi=300, bbox_inches='tight')

plt.close(fig4)

print("Figure 4 saved as Figure_4_GSEA.png")

print("\nAll exploratory analysis scripts have been run and figures saved.")

**Script 2: SHAP Model Interpretability Analysis**

**Filename:** 02_shap_analysis.py

code Python

downloadcontent_copy

expand_less

IGNORE_WHEN_COPYING_START

IGNORE_WHEN_COPYING_END

# =============================================================================

# SCRIPT 02: SHAP MODEL INTERPRETABILITY ANALYSIS

#

# Description:

# This script trains the final SVR Super Model and uses the SHAP library

# to interpret its predictions. It generates and saves the main SHAP summary

# plot, which is a key figure for the manuscript.

#

# Author: [Your Name]

# Date: [Date]

# =============================================================================

# --- Step 1: Import necessary libraries ---

import pandas as pd

import numpy as np

import matplotlib.pyplot as plt

import seaborn as sns

from sklearn.preprocessing import StandardScaler

from sklearn.svm import SVR

from sklearn.pipeline import Pipeline

import shap

print("Libraries imported successfully.")

# --- Step 2: Simulate a comprehensive and realistic dataset ---

def simulate_data_seasonal(n_samples=1000, river_name='River_A'):

# ... (Same simulation function as in script 01)

temp = np.random.uniform(5.0, 30.0, n_samples)

do_saturation = 14.652 - 0.41022 * temp + 0.007991 * temp**2 - 0.000077774 * temp**3

do = do_saturation * np.random.uniform(0.6, 0.95, n_samples)

bod = 5 + (temp / 30) * np.random.uniform(20, 55, n_samples)

cod = bod * np.random.uniform(1.5, 2.5, n_samples)

wqi_simulated = 10 + (do * 1.5) - (np.log1p(bod) * 0.5) - ((temp - 20)**2 * 0.02) + np.random.normal(0, 0.5, n_samples)

df = pd.DataFrame({'DO': do, 'Temp': temp, 'BOD': bod, 'COD': cod, 'River': river_name, 'Predicted_WQI': wqi_simulated})

return df

print("\nSimulating dataset for SHAP analysis...")

df_combined = pd.concat([simulate_data_seasonal(1000), simulate_data_seasonal(1000), simulate_data_seasonal(1000)], ignore_index=True)

print("Simulation complete.")

# --- Step 3: Train the Super Model ---

features_for_model = ['DO', 'Temp', 'BOD', 'COD']

target = 'Predicted_WQI'

X = df_combined[features_for_model]

y = df_combined[target]

super_model = Pipeline([('scaler', StandardScaler()), ('svr', SVR(kernel='rbf', C=100, gamma='auto'))])

print("\nTraining the Super Model...")

super_model.fit(X, y)

print("Model training complete.")

# --- Step 4: Perform SHAP Analysis ---

print("\nPerforming SHAP analysis... (This may take a moment)")

scaler = super_model.named_steps['scaler']

svr_model = super_model.named_steps['svr']

X_scaled = scaler.transform(X)

X_scaled_df = pd.DataFrame(X_scaled, columns=features_for_model)

background_data = shap.sample(X_scaled_df, 100)

explainer = shap.KernelExplainer(svr_model.predict, background_data)

X_sample_scaled = X_scaled_df.sample(n=500, random_state=42)

shap_values = explainer.shap_values(X_sample_scaled)

print("SHAP analysis complete.")

# --- Step 5: Generate and Save SHAP Summary Plot ---

print("\nGenerating Figure 5: SHAP Summary Plot...")

sns.set_theme(style="white", context="talk")

# Use original data for plotting to make feature values interpretable

X_sample_original = scaler.inverse_transform(X_sample_scaled)

X_sample_original_df = pd.DataFrame(X_sample_original, columns=features_for_model)

shap.summary_plot(shap_values, X_sample_original_df, plot_type="dot", show=False)

fig = plt.gcf()

fig.suptitle("Figure 5: SHAP Summary Plot of Feature Impacts", fontsize=18, y=1.0)

ax = plt.gca()

ax.set_xlabel("SHAP value (Impact on model output)")

ax.grid(False)

plt.savefig("Figure_5_SHAP_Summary.png", dpi=300, bbox_inches='tight')

plt.close(fig)

print("Figure 5 saved as Figure_5_SHAP_Summary.png")

print("\nSHAP analysis script has been run successfully.")

**Script 3: Interactive Dash Application**

**Filename:** 03_interactive_dashboard.py

code Python

downloadcontent_copy

expand_less

IGNORE_WHEN_COPYING_START

IGNORE_WHEN_COPYING_END

# =============================================================================

# SCRIPT 03: INTERACTIVE DASHBOARD APPLICATION

#

# Description:

# This script launches a web-based interactive dashboard using Dash.

# The dashboard allows users to dynamically adjust input parameters and see

# the real-time impact on the predicted WQI via a gauge.

# This script is intended for deployment or interactive demonstration.

#

# Author: [Your Name]

# Date: [Date]

# =============================================================================

# --- Step 1: Import necessary libraries ---

import dash

from dash import dcc, html

from dash.dependencies import Input, Output

import plotly.graph_objects as go

import numpy as np

import pandas as pd

from sklearn.preprocessing import StandardScaler

from sklearn.svm import SVR

from sklearn.pipeline import Pipeline

print("Libraries imported successfully.")

# --- Step 2: Simulate and Train the Super Model ---

# (This is needed to create the prediction engine for the dashboard)

def simulate_data(n_samples=500):

do = np.random.uniform(4.0, 12.0, n_samples); temp = np.random.uniform(5.0, 30.0, n_samples)

bod = np.random.uniform(1.0, 60.0, n_samples); cod = bod * np.random.uniform(1.5, 2.5, n_samples)

wqi_target = 10 + (do * 1.5) - (np.log1p(bod) * 0.5) - ((temp - 20)**2 * 0.02) + np.random.normal(0, 0.5, n_samples)

df = pd.DataFrame({'DO': do, 'Temp': temp, 'BOD': bod, 'COD': cod, 'WQI_Target': wqi_target})

return df

df_combined = pd.concat([simulate_data(200), simulate_data(200), simulate_data(200)], ignore_index=True)

features_for_model = ['DO', 'Temp', 'BOD', 'COD']

target = 'WQI_Target'

X = df_combined[features_for_model]; y = df_combined[target]

super_model = Pipeline([('scaler', StandardScaler()), ('svr', SVR(kernel='rbf', C=100, gamma='auto'))])

print("\nTraining the Super Model for the dashboard...")

super_model.fit(X, y)

print("Model training complete.")

average_values = X.mean()

# --- Step 3: Define WQI Status Function ---

def get_wqi_status(wqi_value):

if wqi_value >= 22: return "Excellent"

elif wqi_value >= 18: return "Good"

elif wqi_value >= 14: return "Moderate"

else: return "Poor"

# --- Step 4: Build the Dash Application ---

app = dash.Dash(__name__)

def create_input_block(name, label, min_val, max_val, step, value, marks):

return html.Div([dcc.Checklist(id=f'{name}-switch', options=[{'label': f' Use {label}', 'value': 'ON'}], value=['ON']),

dcc.Slider(id=f'{name}-slider', min=min_val, max=max_val, step=step, value=value, marks=marks)],

style={'paddingTop': '15px'})

app.layout = html.Div(style={'fontFamily': 'Helvetica, Arial, sans-serif', 'maxWidth': '1000px', 'margin': 'auto'}, children=[

html.H1("WQI Prediction Dashboard", style={'textAlign': 'center', 'padding': '20px'}),

html.Div(className='main-container', style={'display': 'flex', 'flexDirection': 'row', 'border': '1px solid #ddd', 'borderRadius': '10px', 'padding': '20px'}, children=[

html.Div(className='left-panel', style={'flex': '1', 'padding': '10px'}, children=[

html.H3("Optional Inputs", style={'textAlign': 'center'}), html.Hr(),

create_input_block('cod', 'COD (mg/l)', 0, 300, 10, 40, {i: str(i) for i in range(0, 301, 100)}),

]),

html.Div(className='center-panel', style={'flex': '2', 'padding': '20px', 'textAlign': 'center'}, children=[

dcc.Graph(id='wqi-gauge'),

html.H2("Current Status:", style={'marginTop': '-50px'}),

html.Div(id='wqi-status-text', style={'fontSize': '32px', 'fontWeight': 'bold'})

]),

html.Div(className='right-panel', style={'flex': '1', 'padding': '10px'}, children=[

html.H3("Essential Inputs", style={'textAlign': 'center'}), html.Hr(),

create_input_block('do', 'DO (mg/l)', 4, 12, 0.5, 8.0, {i: str(i) for i in range(4, 13)}),

create_input_block('temp', 'Temp (°C)', 5, 30, 1, 18, {i: str(i) for i in range(5, 31, 5)}),

create_input_block('bod', 'BOD (mg/l)', 0, 60, 2, 15, {i: str(i) for i in range(0, 61, 20)}),

]),

])

])

# --- Step 5: Define Callbacks ---

@app.callback(

[Output('wqi-gauge', 'figure'), Output('wqi-status-text', 'children')],

[Input(f'{name}-slider', 'value') for name in ['do', 'temp', 'bod', 'cod']] +

[Input(f'{name}-switch', 'value') for name in ['do', 'temp', 'bod', 'cod']]

)

def update_dashboard(do_val, temp_val, bod_val, cod_val, do_on, temp_on, bod_on, cod_on):

input_values = {

'DO': do_val if do_on else average_values['DO'], 'Temp': temp_val if temp_on else average_values['Temp'],

'BOD': bod_val if bod_on else average_values['BOD'], 'COD': cod_val if cod_on else average_values['COD'],

}

input_data = pd.DataFrame([input_values]); predicted_wqi = super_model.predict(input_data)[0]

status_text = get_wqi_status(predicted_wqi)

if status_text == "Excellent": status_color = "#0d6efd"

elif status_text == "Good": status_color = "#198754"

elif status_text == "Moderate": status_color = "#ffc107"

else: status_color = "#dc3545"

fig = go.Figure(go.Indicator(

mode="gauge+number", value=predicted_wqi,

number={'font': {'size': 60}, 'valueformat': '.2f'},

domain={'x': [0, 1], 'y': [0, 1]},

title={'text': f"Predicted WQI<br><span style='font-size:0.8em;color:gray'>{status_text}</span>", 'font': {'size': 24}},

gauge={'axis': {'range': [8, 28]}, 'bar': {'color': status_color, 'thickness': 0.3},

'steps': [{'range': [8, 14], 'color': '#F8F9FA'}, {'range': [14, 18], 'color': '#F8F9FA'},

{'range': [18, 22], 'color': '#F8F9FA'}, {'range': [22, 28], 'color': '#F8F9FA'}],

'threshold': {'line': {'color': "black", 'width': 7}, 'thickness': 1.0, 'value': predicted_wqi}}))

fig.update_layout(height=450, margin=dict(l=10, r=10, t=80, b=10))

status_component = html.Span(status_text, style={'color': status_color})

return fig, status_component

for name in ['do', 'temp', 'bod', 'cod']:

@app.callback(Output(f'{name}-slider', 'disabled'), Input(f'{name}-switch', 'value'))

def toggle_slider(switch_value):

return not switch_value

# --- Step 6: Run the Application ---

if __name__ == '__main__':

# Use jupyter_mode="inline" for Colab/Jupyter, or mode="external" for a local server link.

app.run(jupyter_mode="inline", height=650)
