# Supplementary material for: Water quality index prediction via a robust machine learning model using oxygen-related indices for river water quality monitoring
Source: Sci Rep. 2026 Jan 24;16:6102. doi: 10.1038/s41598-026-36156-3 (PMC12902110; doi:10.1038/s41598-026-36156-3)
Supplement: Supplementary file 3 — Supplementary Material 3 [file 41598_2026_36156_MOESM3_ESM.pdf]

Point Accuracy Metrics

1. **Coefficient of Determination (R^2):** Measures the proportion of the variance in the observed data that is predictable from the model.

   $$R^2 = 1 - [\Sigma (y_i - \hat{y}_i)^2 / \Sigma (y_i - \bar{y})^2] \quad \text{(Eq.S1)}$$

   Where $\bar{y}$ is the mean of the observed data.

2. **Mean Squared Error (MSE):** The average of the squares of the errors. It heavily penalizes large errors.

   $$MSE = (1/n) * \Sigma (y_i - \hat{y}_i)^2 \quad \text{(Eq.S2)}$$

3. **Root Mean Squared Error (RMSE):** The square root of the MSE, expressed in the same units as the predicted variable.

   $$RMSE = \text{sqrt} [(1/n) * \Sigma (y_i - \hat{y}_i)^2] \quad \text{(Eq.S3)}$$

4. **Mean Absolute Error (MAE):** The average of the absolute differences between prediction and actual observation.

   $$MAE = (1/n) * \Sigma|y_i - \hat{y}_i| \quad \text{(Eq.S4)}$$

Relative Error Metrics

1. **Mean Absolute Percentage Error (MAPE):** The mean of the absolute percentage errors.

   $$MAPE = (100/n) * \Sigma|(y_i - \hat{y}_i) / y_i| \quad \text{(Eq.S5)}$$

2. **Symmetric Mean Absolute Percentage Error (SMAPE):** A variation of MAPE that is less biased towards low forecast values.

   $$SMAPE = (100/n) * \Sigma [|\hat{y}_i - y_i| / (|y_i| + |\hat{y}_i|) / 2] \quad \text{(Eq.S6)}$$

3. **Mean Squared Logarithmic Error (MSLE):** Calculates the mean of the squared differences between the natural logarithm of the predicted and actual values.

   $$MSLE = (1/n) * \Sigma (\log (1 + y_i) - \log (1 + \hat{y}_i))^2 \quad \text{(Eq.S7)}$$

Uncertainty and Prediction Interval Metrics

Let L_i and U_i be the lower and upper bounds of the 95% Prediction Interval for the *i*-th prediction, respectively.

1. **Prediction Interval Coverage Probability (PICP):** The percentage of observed values that fall within their 95% prediction intervals.

   $$PICP = (1/n) * \Sigma c_i \quad \text{(Eq.S8)}$$

   where $c_i = 1$ if $L_i \leq y_i \leq U_i$, and $c_i = 0$ otherwise.

2. **Mean Prediction Interval Width (MPIW):** The average width of the prediction intervals. A measure of precision.

   $$MPIW = (1/n) * \Sigma (U_i - L_i) \quad \text{(Eq.S9)}$$

3. **Normalized Mean Prediction Interval Width (NMPIW):** The MPIW normalized by the range of the observed data.

   $$NMPIW = MPIW / (y_{max} - y_{min}) \quad \text{(Eq.S10)}$$

4. **Coverage Width-based Criterion (CWC):** A composite metric that penalizes models with poor coverage (PICP < 95%).

   $$CWC = MPIW * (1 + \gamma * \exp [-\eta (PICP - 0.95)]) \quad \text{(Eq.S11)}$$

   Where $\gamma = 1$ if $PICP < 0.95$ and 0 otherwise, and η is a penalty parameter (e.g., 50).

Probabilistic Metrics

Let F_i be the predicted cumulative distribution function (CDF) for the *i*-th observation, and f_i be the predicted probability density function (PDF). For our Gaussian assumption, these are derived from $N(\hat{y}_i, RMSE)$.

1. **Negative Log-Likelihood (NLL):** Measures the quality of the predicted probability distribution. Lower is better.

   $$NLL = - \Sigma \log(f_i(y_i)) \quad \text{(Eq.S12)}$$

2. **Continuous Ranked Probability Score (CRPS):** A comprehensive metric that generalizes the MAE to probabilistic forecasts.

   $$CRPS = (1/n) * \Sigma \int [F_i(x) - H(x - y_i)]^2 dx \quad \text{(Eq.S13)}$$

   where $H(x)$ is the Heaviside step function.
